# Supplementary material for: Ect2/Pbl Acts via Rho and Polarity Proteins to Direct the Assembly of an Isotropic Actomyosin Cortex upon Mitotic Entry
Source: Dev Cell. 2015 Mar 9;32(5):604–16. doi: 10.1016/j.devcel.2015.01.012 (PMC4359025; doi:10.1016/j.devcel.2015.01.012)
Supplement: Document S1. Supplemental Experimental Procedures and Figures S1–S5 [file mmc1.pdf]

Developmental Cell

Supplemental Information

Ect2/Pbl Acts via Rho and Polarity Proteins to Direct the Assembly of an Isotropic Actomyosin Cortex upon Mitotic Entry

André Rosa, Evi Vlassaks, Franck Pichaud, and Buzz Baum

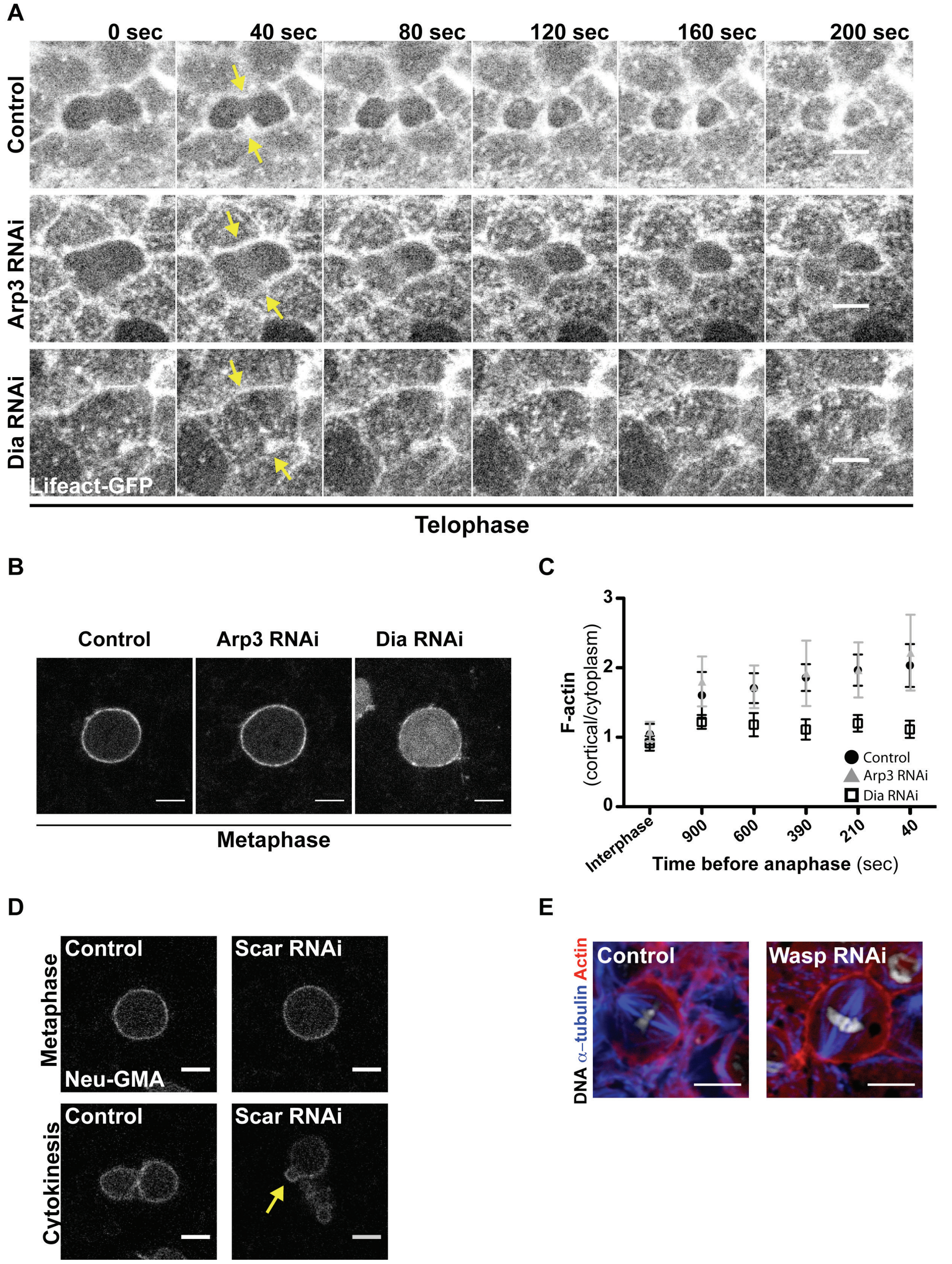

Figure S1. The Arp2/3 complex is not required for the assembly of a mitotic actin cortex; Related to Fig.2. (A) Time-lapse shows the apical portion of a cell expressing UAS-Lifeact::GFP together with *arp3* or *dia* dsRNA imaged in the plane of the epithelium. Time T=0 sec indicates the

onset of cytokinesis. Yellow arrow points to furrowing in control and Arp3 RNAi cells and to the equivalent region of Dia RNAi cells. **(B)** Metaphase cells viewed in the plane of the epithelium expressing UAS-GMA (control) to label F-actin together with Arp3 or Dia RNAi. **(C)** Graph indicates the cortical/cytoplasm ratio of F-actin intensity in the spindle plane as control, Arp3 and Dia RNAi cells enter mitosis. (Mean±SD, N≥25 cells from at least 3 different pupae). **(D)** The cortex of control (Neu:GMA) and SCAR RNAi cells in mitosis and following mitotic exit imaged at the level of the spindle in xy. **(E)** Control and Wasp RNAi cells imaged in the plane of the epithelium stained for filamentous actin (red), DNA (white) and α-tubulin (blue). Scale bar: 5 μm.

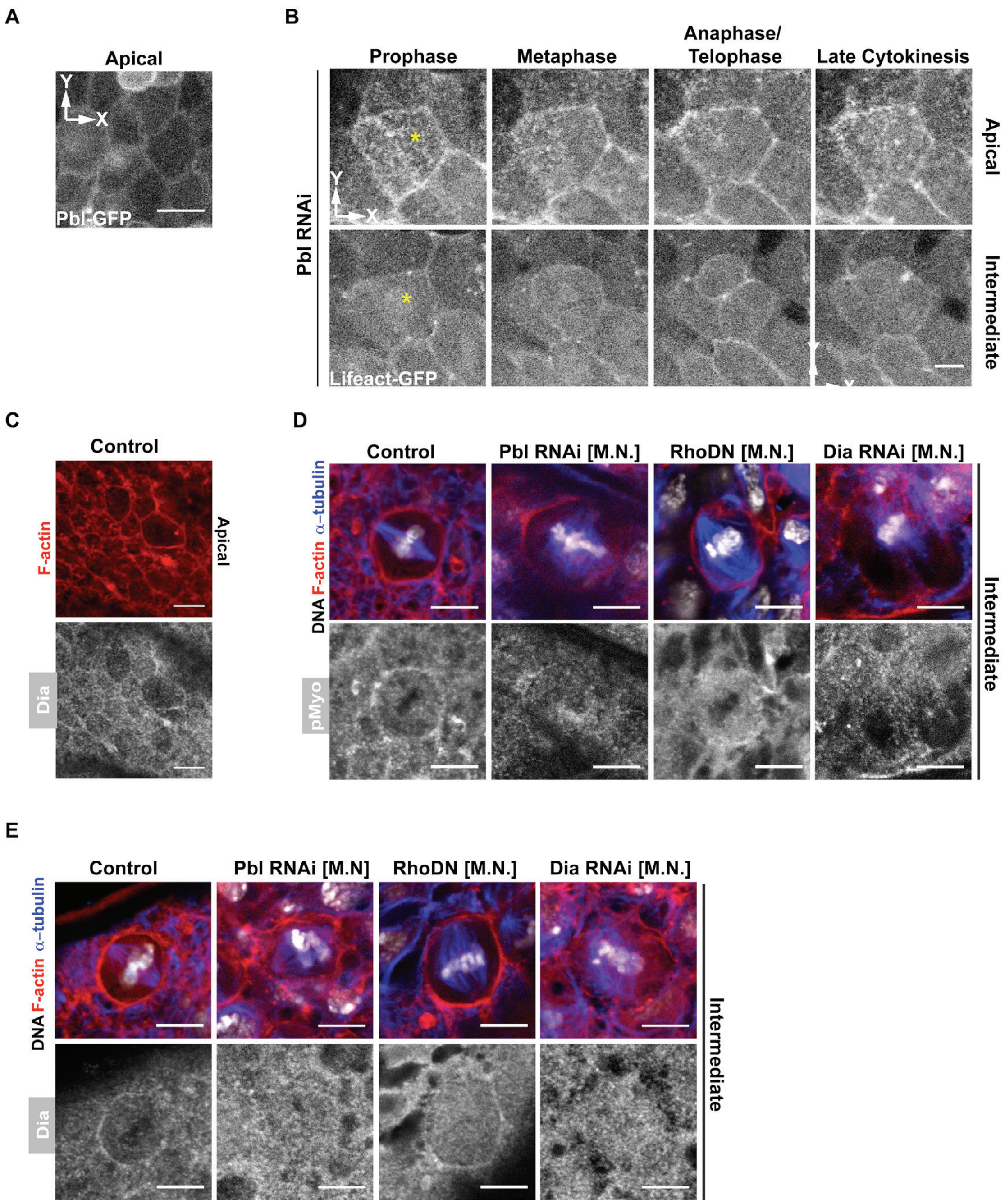

Figure S2. Pbl/Ect2 is required for the correct cortical localization of Dia and Myosin-II; Related to Fig.3. (A) An apical section showing interphase epithelial cells expressing low levels of UAS-Pbl::GFP imaged in the plane of the epithelium. **(B)** Characteristic hypomorphic Pbl RNAi phenotype in a cell labelled with UAS-Lifeact::GFP (as seen in Figure 3) imaged at apical and intermediate sections in the plane of the epithelium. Yellow asterisk indicates a dividing cell. **(C)** Apical section of tissue to show the localization of F-actin (red) and Diaphanous (white) during interphase. **(D)** F-actin (red), DNA

(white), α-tubulin (blue) and phospho-Myosin-II (white) in control, Pbl RNAi, Dia RNAi and Rho1.N19 expressing cells at metaphase. **(E)** As for D, but with Diaphanous shown in white. M.N. = Multinucleated. Scale bar: 5 μm.

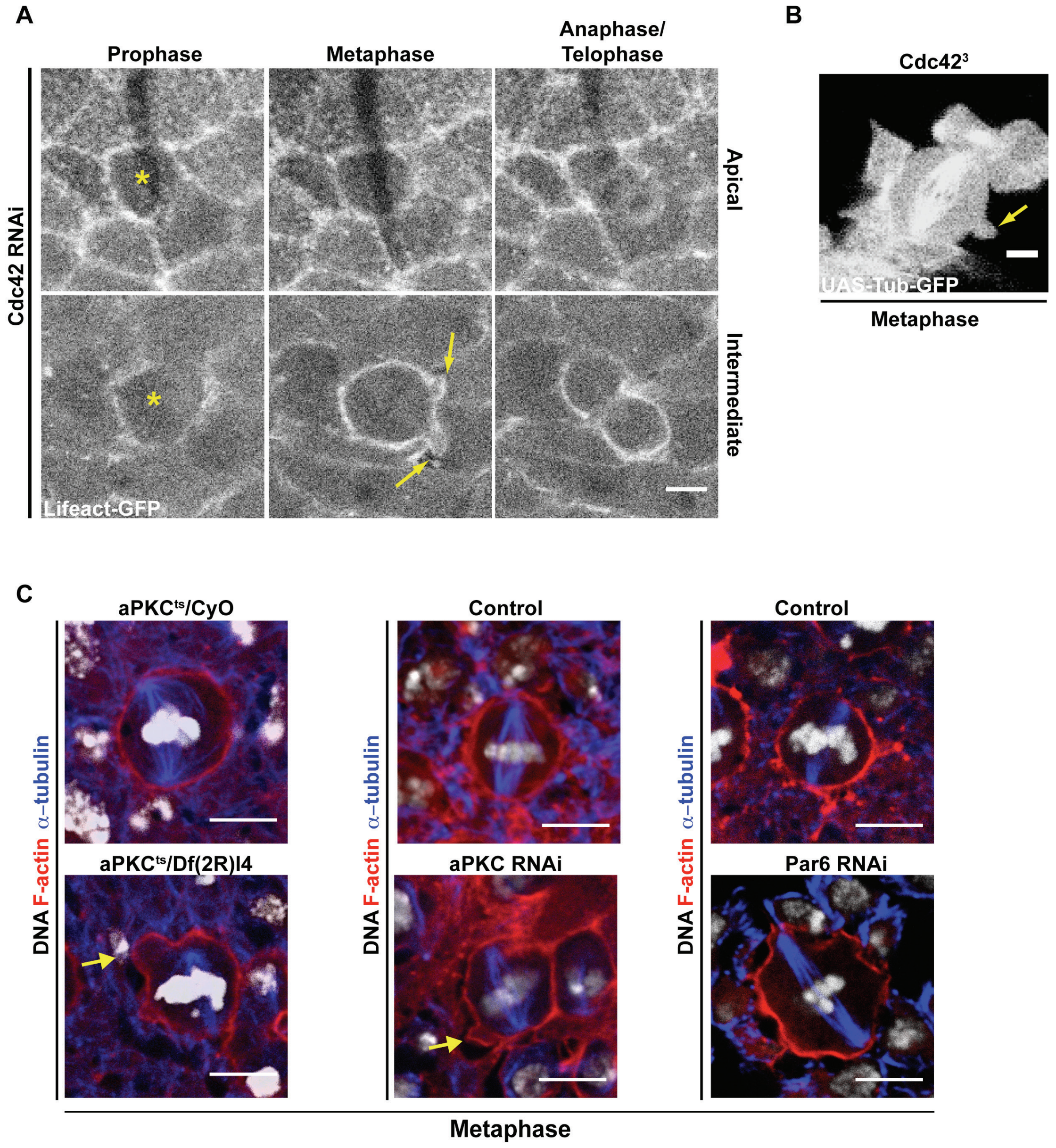

Figure S3. Cdc42, aPKC and Par6 control actin cortex stability; Related to Fig.4. (A) Apical and spindle-level (intermediate) views in the plane of the epithelium for Cdc42 RNAi cells expressing UAS-Lifeact::GFP at different stages of mitosis (as seen in Figure 4). Yellow asterisk indicates dividing cell. Yellow arrows indicate blebbing. **(B)** Mosaic clone of marked *cdc42*^{3} cells expressing UAS-Tub::GFP viewed in xy. Yellow arrow indicates metaphase bleb. **(C)** Control, aPKC RNAi, Par6 RNAi and homozygous *aPKC*^{ts} mutant cells in metaphase labelled to show F-actin (red), DNA (white) and α-tubulin (blue). Yellow arrows indicate cell shape deformations. Scale bar: 5 μm.

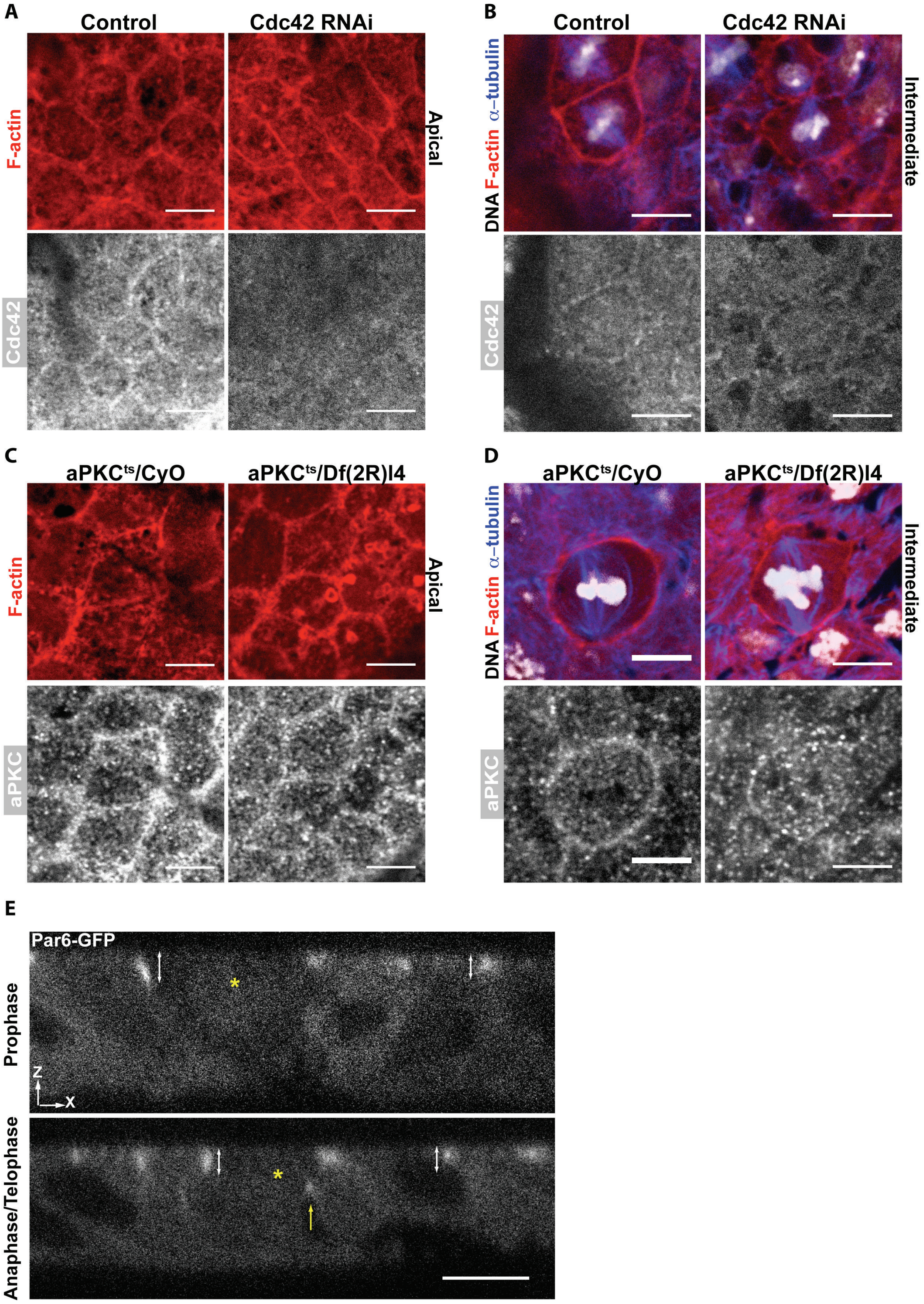

Figure S4. Lateral spreading of Cdc42, aPKC and Par6 during mitosis; Related to Fig.5. (A) Cdc42 RNAi cells labelled for F-actin (red) and using anti-Cdc42 antibody (white) imaged in xy at an apical plane of the epithelium. **(B)** A mitotic Cdc42 RNAi cell stained for F-actin (red), DNA (white), α-tubulin (blue) and Cdc42 (white). **(C)** Control *(aPKC^{ts}/CyO)* and homozygous *aPKC^{ts}*

mutant tissue *(aPKC^{ts}/Df(2R)l4)* labelled for F-actin (red) and aPKC (white) and imaged in xy at the apical plane of the tissue. **(D)** As in C but stained for F-actin (red), DNA (white), α-tubulin (blue) and aPKC (white) to show mitotic cells in xy at the spindle plane. **(E)** Live imaging of a cell expressing Par6-GFP at prophase and anaphase/telophase in cross-section (xz). White double arrow marks polarized interphase Par6::GFP. Yellow asterisk marks dividing cell. Yellow arrow marks cleavage furrow. Scale bar: 5μm.

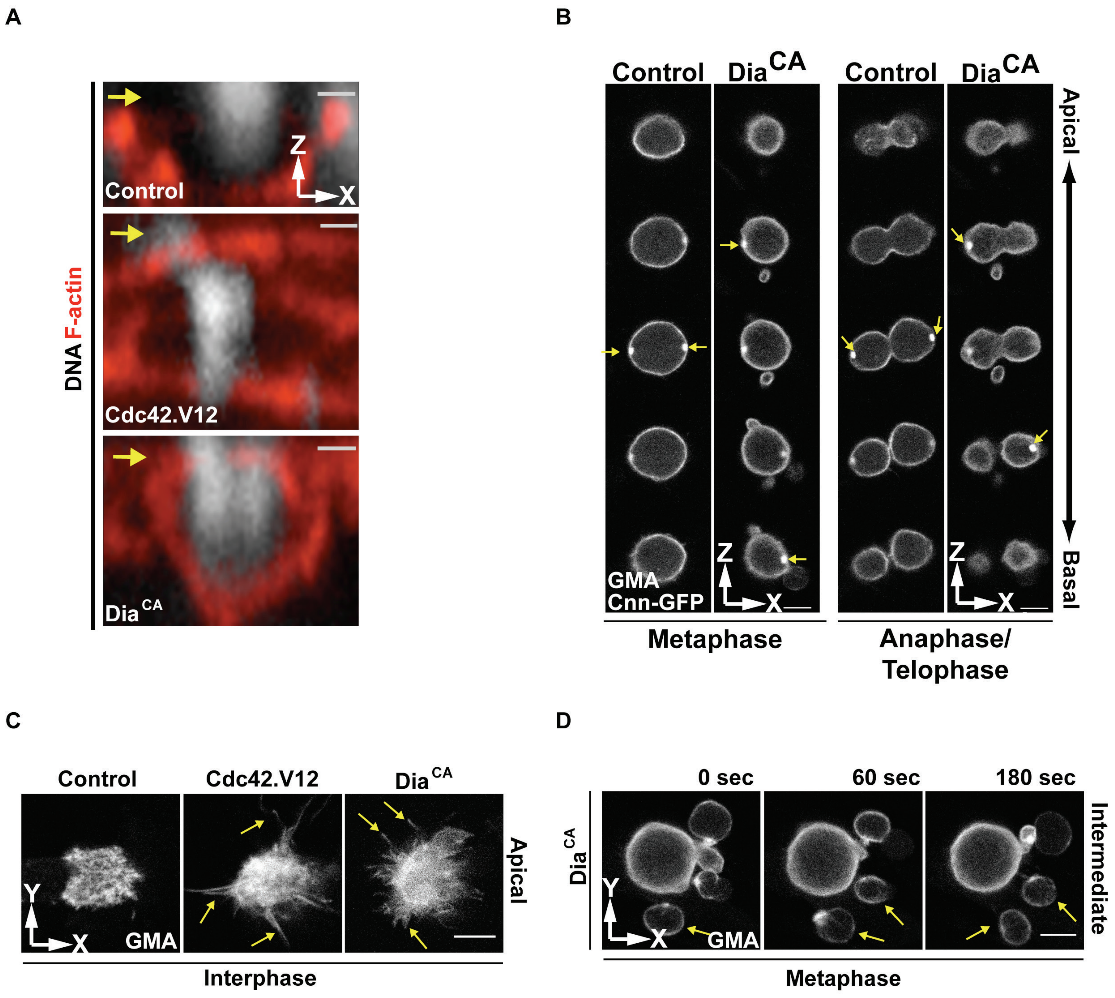

Figure S5. Constitutively active forms of Cdc42 and Dia are sufficient to drive apical filament formation in mitosis; Related to Fig.6. (A) Cross section of control, Cdc42.V12 and Dia^{CA} expressing cells in metaphase labelled for F-actin (red) and DNA (white). Note apical actin-rich cortex in Cdc42.V12 and Dia^{CA} cells (yellow arrow). Scale bar: 1.3 µm. **(B)** Confocal sections of plane of the tissue (xy) taken from apical to basal for control and Dia^{CA} expressing SOP cells marked with UAS-GMA::GFP and UAS-CNN::GFP in metaphase and telophase. Yellow arrows mark centrosomes. Scale bar: 5 µm. **(C)** Apical section in plane of epithelium (xy) of actin in control, Cdc42.V12 and Dia^{CA} expressing SOP cells in interphase marked with UAS-GMA::GFP. Yellow arrows mark actin-rich protrusions. Scale bar: 5 µm. **(D)** Time-lapse movie of a mitotic Dia^{CA} expressing SOP cell imaged in xy at the level of the spindle using UAS-GMA::GFP. Yellow arrows mark blebs that have been pinched off. Scale bar: 5 µm.

Supplemental Experimental Procedures

Fly Stocks

RNAi lines used to silence the expression of the following genes: *cdc42* (NIG-Fly library, ID 12530R-3), *aPKC* (VDRC-Fly library, ID 105624), *arp3* (VDRC-Fly library, ID 35260), *diaphanous* (VDRC-Fly library, ID 103914), *pebble* (VDRC-Fly library, ID 109305), *par-6* (VDRC-Fly library, ID 19731).

Dissections and live imaging

The expression of Neu-GMA was used to label actin filaments in P1 cells (Cohen et al., 2010); UAS-Tub::RFP was expressed from the *pnr* promoter to label microtubules in epithelial cells; UAS-Lifeact::GFP was expressed from the *pnr* promoter to label actin filaments in epithelial cells. The Neu-Gal4 driver was used to express UAS-CNN::GFP, UAS-GMA and UAS-Dia^{CA} in P1 cells. RNAi-induced gene silencing was accomplished by using the *pnr*-Gal4 driver to express Gal4-responsive hairpin dsRNAs in transgenic flies (Mummery-Widmer et al., 2009). In case of RhoDN, Cdc42.V12 and Dia^{CA} transgenes and where the RNAi transgenes were toxic, the Gal80ts system was used to limit expression, after which flies were shifted to 29°C at 6-9 hours AP (Zeidler et al., 2004).

apkc^{ts} zygotic mutant pupae were obtained by crossing the *apkc*^{ts}/CyO, pAct5C-GFP stock with the Df(2R)l4/CyO, pAct5C-GFP and pupae were selected by the absence of GFP, whereas GFP-positive larvae were used as controls.

The following antibodies and dyes were used at the indicated dilutions for our study: Guinea Pig anti-Par6 1:500 (gift from Frank Pichaud); Rabbit anti-

aPKCζ (c-20) 1:100 (Santa Cruz Biotechonology); Rabbit pMyosin Light Chain II (S19) 1:30 (Cell Signaling Technology); Mouse anti-α-tubulin (clone DM1A) 1:250 (Sigma); Rabbit anti-Cdc42 1:20 (Eurogentec); Chicken anti-GFP 1:500 (Abcam); rabbit anti-V5 1:100 (Abcam); Rabbit anti-Dia 1:400 (Gift from Steven A. Wasserman); TRITC-conjugated Phalloidin 1:500; DAPI 1:1000. Secondary antibodies from Molecular Probes were labeled with Alexa 488, 546 and 647 dyes.

Expression vectors, Cell culture and Immunoprecipitation

The Par6 and Pbl cDNA constructs were generated through the Gateway system (Invitrogen) using full-length Drosophila Par-6 and Pbl cDNA as template. Fragments were inserted in frame into the pAct5C vector (DGRC, Indiana University) to create C-terminal Flag-tagged and N-terminal HA-tagged constructs respectively. For transfection, cells were seeded at $2x10^6$ cells / 600 μl in a 12 well plate and allowed to adhere overnight.

48h after transfection (Effectene, Qiagen), whole cell lysates were washed once with PBS and homogenized in lysis buffer (50mM Tris pH7.5, 150mM NaCl, 1% Triton X-100, 1mM EDTA) containing protease (Roche) and phosphatase (Sigma) inhibitor cocktail. Following centrifugation, 500 μg of supernatant was immunoprecipitated with 5 μg of antibody (rabbit anti-HA [abcam], rat anti-HA [Roche] or mouse anti-flag [Sigma]) in 500 μl lysis buffer overnight at 4°C on a rotator. Protein A/G magnetic beads (Pierce, Thermo Scientific) were added, and the mixture was incubated for an additional 1h at 4°C. Beads/extracts were washed three times with BSA/lysis buffer solution followed by three washes in lysis buffer. Bound proteins were eluted from the

beads in 2x Laemmli buffer by boiling for 10 min before separation by SDS-PAGE. For Western Blot analysis, gels were blotted to a polyvinylidene difluoride membrane. Blots were then incubated in 5% milk for 30 min in PBS-0.1%Tween-20, in primary antibody (rabbit anti-HA [abcam], mouse anti-flag [Sigma] or rabbit anti-aPKC [Sigma]) overnight at 4°C, and finally in secondary antibody for 1h.

In vitro binding assay

Recombinant GST::Cdc42 was produced in BL21-A1 *E. coli* by using the pDEST-15 vector (Invitrogen). 4 h after induction with 0.2% L-arabinose, cells were pelleted and resuspended in GST lysis buffer [50 mM Tris–HCl pH 7.6, 50 mM NaCl, 5mM $MgCl_2$, 10 mM dithiothreitol (DTT), protease inhibitor cocktail Complete (Roche)]. Cells were sonicated and debris was removed by centrifugation at 9000 RPM for 15 min. Glutathione–Sepharose beads were incubated with the supernatant on a rotator for 30 min at 4°C. Beads were washed 5 times with GST storage buffer [50 mM Tris–HCl pH 7.6, 50 mM NaCl, 5mM $MgCl_2$, 1 mM dithiothreitol (DTT), protease inhibitor cocktail Complete (Roche)], and stored in the same buffer. Production and purification of GST::Cdc42 proteins were verified by Coomassie staining of SDS/PAGE gel. GST-pull-down assays were performed by using 300 µl of Myc::Pbl transfected (or salmon sperm transfected as negative control) S2 cell lysates along with 5-30 µl of glutathione-Sepharose beads with bound GST-Cdc42. After 1h incubation with rotation at 4°C, the supernatant was removed and the beads were washed five times with lysis buffer (see immunoprecipitation for recipe). After SDS/PAGE, Pbl and recombinant GST proteins were detected

by Western blotting using anti-Myc (Santa Cruz) and anti-GST (Sigma) antibody.

References

Cohen, M., Georgiou, M., Stevenson, N.L., Miodownik, M., and Baum, B. (2010). Dynamic filopodia transmit intermittent Delta-Notch signaling to drive pattern refinement during lateral inhibition. Developmental cell *19*, 78-89.

Mummery-Widmer, J.L., Yamazaki, M., Stoeger, T., Novatchkova, M., Bhalerao, S., Chen, D., Dietzl, G., Dickson, B.J., and Knoblich, J.A. (2009). Genome-wide analysis of Notch signalling in Drosophila by transgenic RNAi. Nature *458*, 987-992.

Zeidler, M.P., Tan, C., Bellaiche, Y., Cherry, S., Hader, S., Gayko, U., and Perrimon, N. (2004). Temperature-sensitive control of protein activity by conditionally splicing inteins. Nature biotechnology *22*, 871-876.
