# Supplementary material for: Ect2/Pbl Acts via Rho and Polarity Proteins to Direct the Assembly of an Isotropic Actomyosin Cortex upon Mitotic Entry
Source: Dev Cell. 2015 Mar 9;32(5):604–16. doi: 10.1016/j.devcel.2015.01.012 (PMC4359025; doi:10.1016/j.devcel.2015.01.012)
Supplement: Document S2. Article plus Supplemental Information [file mmc2.pdf]

Article

Developmental Cell

Ect2/Pbl Acts via Rho and Polarity Proteins to Direct the Assembly of an Isotropic Actomyosin Cortex upon Mitotic Entry

Highlights

- Pbl/Ect2 drives a shift in epithelial polarity upon entry into mitosis
- Lateral spreading of Cdc42/aPKC/Par6 aids assembly of an isotropic metaphase cortex
- Mitosis triggers a switch from Arp2/3 to Dia-mediated cortical actin nucleation

Authors

André Rosa, Evi Vlassaks, Franck Pichaud, Buzz Baum

Correspondence

b.baum@ucl.ac.uk

In Brief

Rosa et al. identify a pathway activated upon entry into mitosis that drives lateral spreading of "apical" polarity proteins to coordinate the remodeling of cell shape and cytoskeleton. This mitotic repurposing of polarity proteins generates the rigid rounded cortex required for successful division in a crowded tissue.

CrossMark

Rosa et al., 2015, Developmental Cell *32*, 604–616
March 9, 2015 ©2015 The Authors
http://dx.doi.org/10.1016/j.devcel.2015.01.012

CellPress

Developmental Cell
Article

Ect2/Pbl Acts via Rho and Polarity Proteins to Direct the Assembly of an Isotropic Actomyosin Cortex upon Mitotic Entry

André Rosa,[1,2] Evi Vlassaks,[1] Franck Pichaud,[1] and Buzz Baum[1,*]
[1]MRC Laboratory of Molecular Cell Biology, UCL, Gower Street, London WC1E 6BT, UK
[2]Graduate Program in Areas of Basic and Applied Biology (GABBA), University of Porto, 4200-465 Porto, Portugal
*Correspondence: b.baum@ucl.ac.uk
http://dx.doi.org/10.1016/j.devcel.2015.01.012
This is an open access article under the CC BY license (http://creativecommons.org/licenses/by/4.0/).

SUMMARY

Entry into mitosis is accompanied by profound changes in cortical actomyosin organization. Here, we delineate a pathway downstream of the RhoGEF Pbl/Ect2 that directs this process in a model epithelium. Our data suggest that the release of Pbl/Ect2 from the nucleus at mitotic entry drives Rho-dependent activation of Myosin-II and, in parallel, induces a switch from Arp2/3 to Diaphanous-mediated cortical actin nucleation that depends on Cdc42, aPKC, and Par6. At the same time, the mitotic relocalization of these apical protein complexes to more lateral cell surfaces enables Cdc42/aPKC/Par6 to take on a mitosis-specific function—aiding the assembly of a relatively isotropic metaphase cortex. Together, these data reveal how the repolarization and remodeling of the actomyosin cortex are coordinated upon entry into mitosis to provide cells with the isotropic and rigid form they need to undergo faithful chromosome segregation and division in a crowded tissue environment.

INTRODUCTION

As animal cells enter mitosis they undergo profound changes in cell shape that are driven by the dynamic remodeling of the actomyosin cortex (Kunda and Baum, 2009). The mitotic cortex has been shown to perform a number of important functions. It helps to ensure timely centrosome separation (Rosenblatt et al., 2004), provides cells with a rigid protective shell in which to assemble a mitotic spindle (Carreno et al., 2008; Kunda et al., 2008; Lancaster et al., 2013), guides spindle orientation (Fink et al., 2011; Luxenburg et al., 2011; Théry et al., 2005), and helps to set the stage for cytokinesis (Kunda et al., 2012; Matthews et al., 2012; Sedzinski et al., 2011). The forces generated during mitotic rounding are considerable and sufficient to drive tissue buckling (Kondo and Hayashi, 2013).

While the nucleators required for mitotic actin filament assembly remain unclear (Bovellan et al., 2014), a number of regulators have been identified that contribute to remodeling of the actomyosin cortex at mitotic entry. In cell culture, these include activation of Ect2/Pbl, which acts via RhoA and Myosin-II (Cramer and Mitchison, 1997; Maddox and Burridge, 2003; Matthews et al., 2012) to initiate mitotic rounding, and ERM proteins, which crosslink F-actin to the overlying plasma membrane. Together, these molecular changes generate a relatively isotropic and stiff actin-based cortex (Carreno et al., 2008; Kunda et al., 2008) that, in combination with osmotic pressure (Stewart et al., 2011) and the disassembly of stress fibers and focal contacts (Dao et al., 2009), give mitotic cells their characteristic rigid and rounded form.

Cells dividing in an epithelium face additional challenges. Cell-cell junctions must be maintained to avoid division compromising the integrity of the tissue. Moreover, cells must generate rounding forces large enough to deform surrounding cells in order to make room for the developing spindle (Lancaster et al., 2013; Luxenburg et al., 2011; Nakajima et al., 2013). Accordingly, an epithelial cell undergoing symmetrical division rounds up to the apical surface as it enters mitosis (Reinsch and Karsenti, 1994). This enables the cell to maintain its apically positioned adherens junctions (AJs) (Founounou et al., 2013; Guillot and Lecuit, 2013; Herszterg et al., 2013; Reinsch and Karsenti, 1994), to assemble a relatively isotropic actin-based cortex, and to align its spindle along the plane of the epithelium (Lu et al., 2001; Luxenburg et al., 2011; Nakajima et al., 2013), before dividing in two.

Here, to characterize the changes in the polarized organization of the actin cytoskeleton that accompany mitotic entry in the context of an epithelium, we studied symmetrical epithelial cell divisions within the fly notum. We find that the assembly of a mechanically stable metaphase cortex depends on the broad specificity RhoGEF Pbl/Ect2, which induces a lateral shift in the distribution of the polarity regulators Cdc42, aPKC, and Par6, leading to the assembly of a relatively isotropic Diaphanous-dependent actomyosin cytoskeleton, as required for mitosis and cell division in a crowded tissue environment.

RESULTS

The Actomyosin Cortex Is Remodeled as Epithelial Cells Enter Mitosis and Round Up

To better understand the coupling between changes in cell morphology and actin remodeling when epithelial cells enter mitosis, we followed cell divisions within the developing fly notum using confocal time-lapse microscopy (Bosveld et al., 2012;

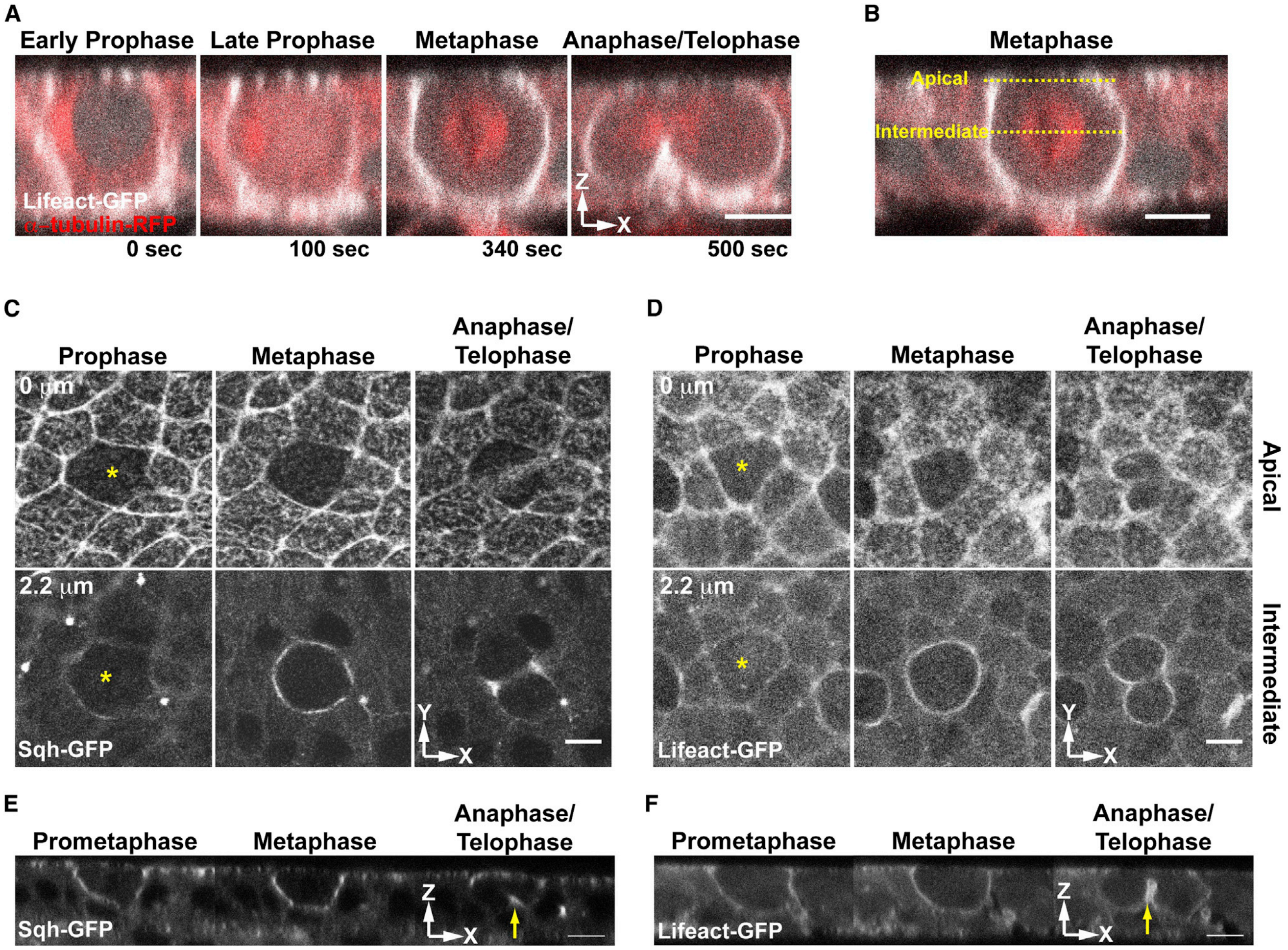

Figure 1. Actin and Myosin Accumulate at the Cell Cortex as Cells Enter Mitosis

(A) Time-lapse of a dividing cell in cross-section (xz) expressing Lifeact::GFP and Tub::RFP. Time T = 0 s onset prophase; T = 500 s anaphase/telophase.

(B) Similar view of a metaphase cell expressing Lifeact::GFP and Tub::RFP. Yellow dashed lines mark apical and intermediate sections.

(C) Apical and intermediate sections (xy) in plane of Sqh::GFP expressing epithelium.

(D) Apical and intermediate views (xy) of a Lifeact::GFP expressing notum. Yellow asterisks mark dividing cells.

(E and F) Time-lapse of (E) a mitotic cell expressing Sqh::GFP in cross-section and (F) a mitotic cell expressing UAS-Lifeact::GFP. Yellow arrow: cleavage furrow. Scale bars, 5 μm.

Marinari et al., 2012). Lifeact::GFP and RFP::Tubulin were expressed under the control of the *pannier* (*pnr*) driver to visualize cortical remodeling, nuclear envelope permeabilization, and spindle assembly. In parallel, a Squash::GFP gene-trap line was used to label Myosin-II. Using these markers, epithelial cells were seen rounding in prophase. As they rounded, cells accumulated cortical F-actin and Myosin-II (Figures 1A, 1C, and 1D) and lost the apical mesh of medial F-actin and Myosin-II (Figures 1C–1F: compare mitotic cell marked with yellow asterisk with neighboring interphase cells). By metaphase, cells had established a stable actomyosin-rich lateral cortex and had adopted a near-spherical shape (Figures 1A–1F, metaphase panels). Then, at mitotic exit, actomyosin was first seen accumulating at the basal cortex. As previously reported (Founounou et al., 2013; Guillot and Lecuit, 2013; Herszterg et al., 2013; Reinsch and Karsenti, 1994), this contractile actomyosin ring moved apically to divide cells into two (Figures 1A and 1C–1F, anaphase/telophase panels). Finally, in the period between the completion of division and the relaxation of cells back into the epithelium, levels of F-actin remained high around the cortex, despite the relocalization of Myosin-II to the cleavage furrow (Figures 1A, 1D, and 1F, anaphase/telophase panels). This series of events is broadly similar to that previously described for many other systems (Cramer and Mitchison, 1997; Reinsch and Karsenti, 1994; Zang et al., 1997), reinforcing the notion that it is a highly conserved process that is likely to be governed by conserved actin regulators.

Next, to identify the molecular mechanisms involved we focused our attention on the two best understood actin nucleators: the Arp2/3 complex and Dia (Castrillon and Wasserman, 1994).

Dia Is Required for Assembly of the Mitotic Actin Cortex

To test the mitotic functions of the Arp2/3 complex and Dia, we used RNAi to silence the expression of *arp3* and *dia*. The

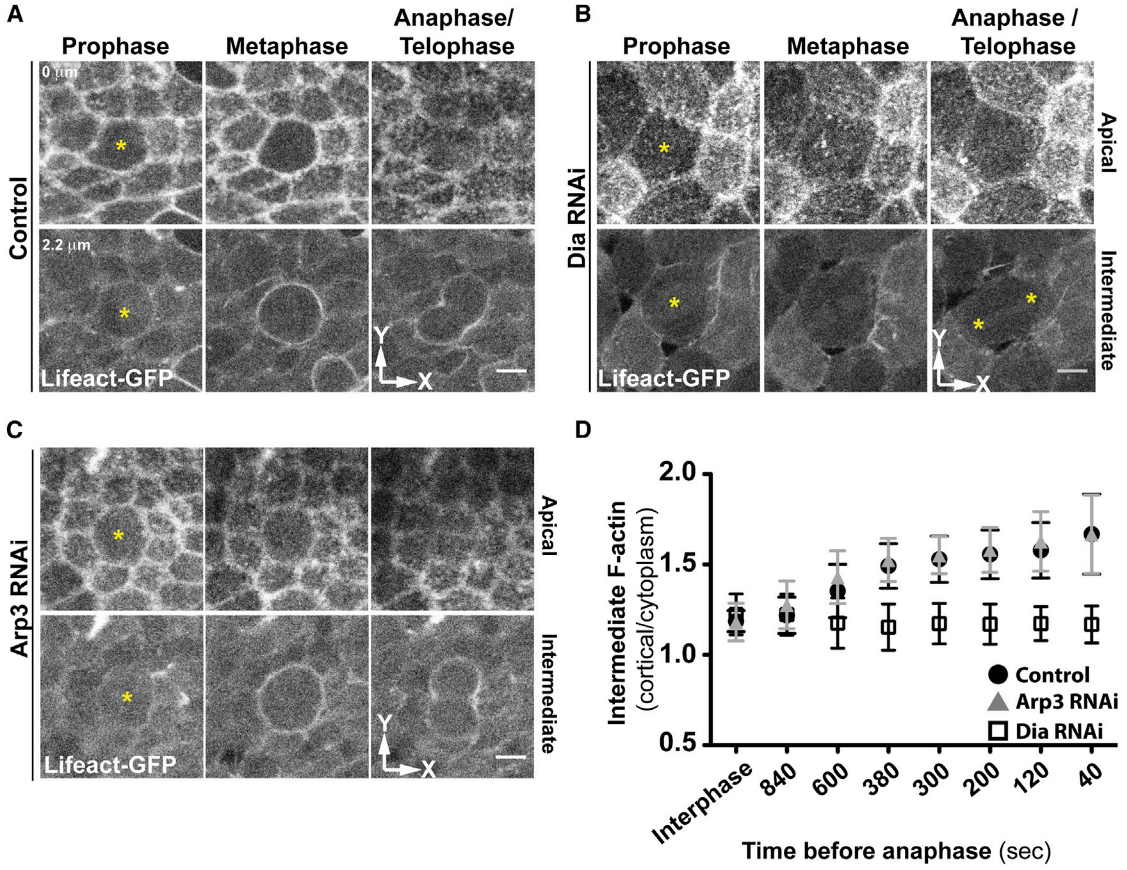

Figure 2. Diaphanous Is Required for the Formation of the Mitotic Actin Cortex

(A–C) Apical and intermediate views (xy) of Lifeact::GFP in control (A), Dia RNAi (B), and Arp3 RNAi epithelia (C). Yellow asterisks mark mitotic cells. Scale bars, 5 μm.

(D) Comparison of cortical/cytoplasmic filamentous actin at several time points before anaphase for control, Arp3, and Dia RNAi conditions. (Mean ± SD, n ≥ 30 cells from at least 3 different pupae.)

See also Figure S1.

assembly of the mitotic F-actin cortex, visualized by the presence of UAS-Lifeact::GFP (Riedl et al., 2008), was unaffected by the expression of Arp3 double-stranded RNA (dsRNA) (Figure 2C). Thus, as Arp3 RNAi cells progressed through mitosis, they were nearly indistinguishable from control cells (compare Figure 2A with Figure 2C) and in every case successfully completed cytokinesis (Figure S1A). Because cells exhibited two previously described loss-of-function phenotypes, it is unlikely that this was due to a failure in the RNAi-mediated Arp3 knockdown: (1) Arp3 silencing prevented the accumulation of F-actin at the new cell interface following division (Herszteg et al., 2013) (Figure S1A) and (2) caused defects in basal protrusions in interphase (Georgiou and Baum, 2010). Moreover, silencing of either of the two key cortical Arp2/3 activators, SCAR or Wasp, had no effect on assembly of the mitotic F-actin cortex (Figures S1D and S1E). By contrast, Dia silencing had a profound effect on the integrity of the mitotic cell cortex and on mitotic cell shape (Figure 2B). It induced a nearly complete loss of cortical F-actin in metaphase cells, which was followed by a failure in cytokinesis, confirming the well-established role for Dia in the formation and contraction of the actomyosin ring (Castrillon and Wasserman, 1994; Grosshans et al., 2005). As a quantitative measure of the dynamic mitotic cortex assembly in each case, we measured the ratio of mean values of cortical/cytoplasmic UAS-Lifeact::GFP (see Experimental Procedures) in a central confocal plane within control, Arp3, and Dia RNAi cells. Both control and Arp3 RNAi cells exhibited a steady 1.7-fold increase in the accumulation of F-actin at the cell cortex following mitotic entry. In nota expressing Dia dsRNA, however, cortical F-actin levels remained close to those seen in interphase (Figure 2D). These data were confirmed when the analysis was replicated in asymmetrically dividing sensory organ precursor cells (SOP) (Figures S1B and S1C), which can be studied without the confounding effects of cortical GFP signals from neighboring interphase cells. Together, these data identify Dia as the primary actin nucleator driving the assembly of the mitotic cortex during both symmetric and asymmetric divisions.

Pbl Acts Upstream of Rho1 and Dia in the Assembly of a Mitotic Actin Cortex

In animal cells, the actomyosin ring is assembled in response to the local accumulation (Fededa and Gerlich, 2012) and activation

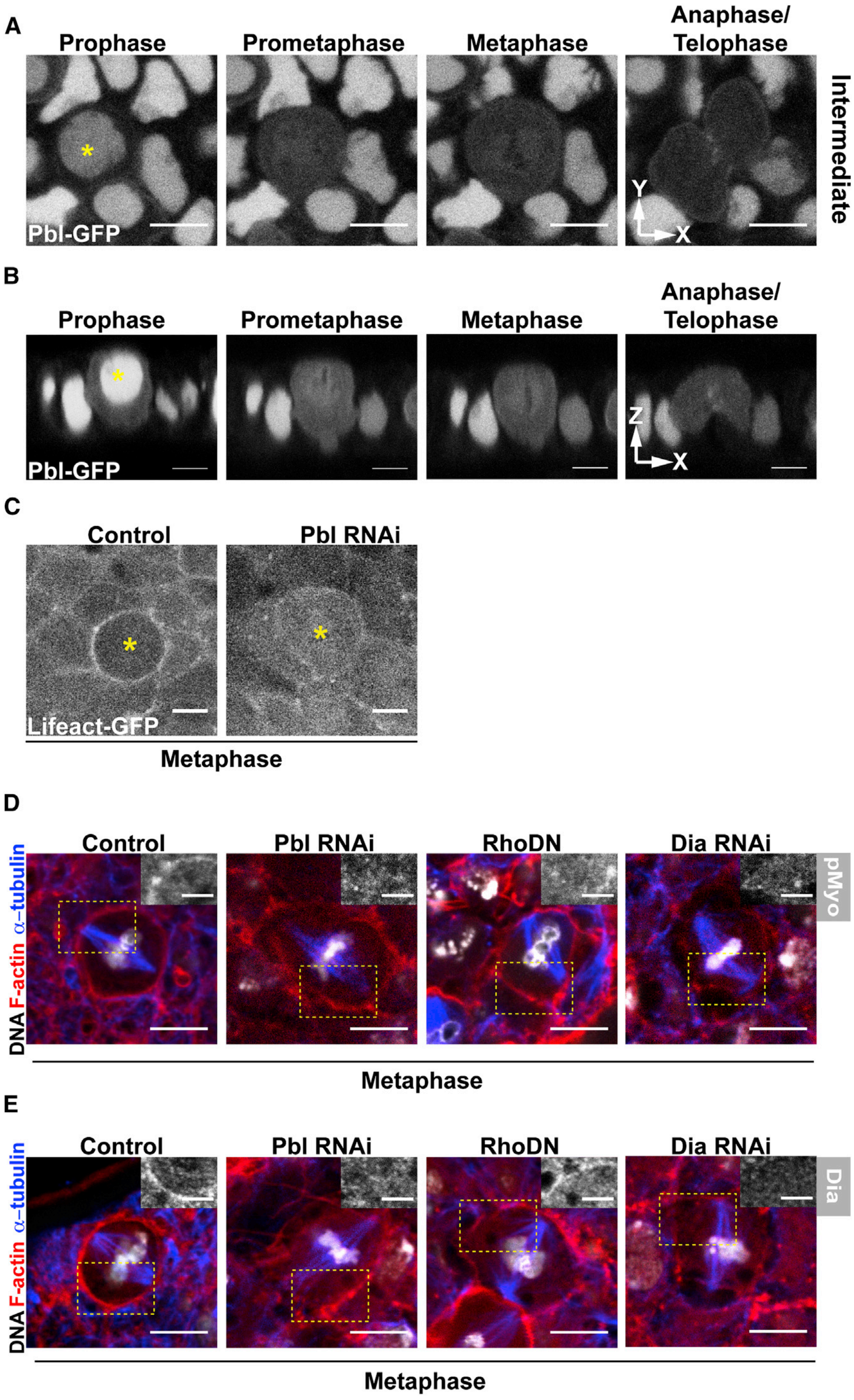

Figure 3. Pebble Is Required for the Correct Cortical Localization of Dia and Formation of an Actin Cortex

(A) Intermediate section (xy) of Pbl::GFP expressing notum. Yellow asterisk marks a dividing cell.

(B) Cross-section (xz) of Pbl::GFP expressing dividing cell (yellow asterisk).

(C) Intermediate section in plane of epithelium (xy) of Lifeact::GFP labeled mitotic cells in control and Pebble RNAi tissue.

(D and E) Similar view of metaphase cells in control (*pnr*-Gal4), Pbl RNAi, or Dia RNAi epithelia, and in tissue expressing Rho1.N19 (DN), stained for F-actin (red), DNA (white), and α-tubulin (blue), together with (D) phospho-Myosin-II (Inset, white) or (E) Diaphanous (Inset, white).

Scale bars, 5 μm. See also Figure S2.

(Petronczki et al., 2007) of the guanine nucleotide exchange factor, Pbl/Ect2, at the spindle midzone. This leads to the local activation of Rho1 GTPase and its downstream effectors Dia (O'Keefe et al., 2001; Prokopenko et al., 1999), together with Rok and Myosin-II. More recently, Ect2 (the human ortholog of Pbl), RhoA, and Myosin-II were shown to have an additional function in mitotic rounding and the assembly of a stiff metaphase actin cortex (Maddox and Burridge, 2003; Matthews et al., 2012). Thus, in human cells, a similar pathway appears to drive assembly of the actomyosin cortex upon mitotic entry and the actin-based cytokinesis ring at mitotic exit. Accordingly, Pbl and Rho1 appeared to be good candidates as regulators of mitotic cortical assembly in *Drosophila* epithelial cells.

To test this idea, we began by looking at the dynamic localization of a GFP-tagged version of Pbl (van Impel et al., 2009) (expressed within the *pnr* domain) during passage through mitosis. This fusion construct is known to rescue *pbl* cytokinetic defects (Zavortink et al., 2005). In interphase cells, the bulk of Pbl was localized to the nucleus. In addition, a small pool of the fusion protein was found at the AJs (Figure S2A). Upon entry into mitosis, bulk Pbl moved into the cytoplasm (Figures 3A and 3B), with a proportion accumulating around the lateral cortex. Finally, at mitotic exit, Pbl-GFP became recruited to the spindle midzone as previously described (Somers and Saint, 2003; Zavortink et al., 2005). This dynamic pattern of Pbl relocalization during mitotic progression in a developing epithelium is similar to that described for human cells (Matthews et al., 2012). When dsRNA was used to deplete Pbl within the *pnr* domain, we observed a reduction in the density of F-actin at interphase cell-cell junctions, and a loss of the actin-rich metaphase cortex and the cytokinesis ring in both live (Figures 3C and S2B) and fixed (Figures 3D, 3E, S2D, and S2E) preparations. Since this phenotype was similar to that seen following Dia RNAi, it suggested a role for Pbl/Ect2 in controlling the local activation of Dia. To test this hypothesis we made use of a Dia antibody, whose specificity was confirmed using RNAi (Figures 3E, 4E, and S2E). In interphase, Dia was found at apical cell-cell junctions (Figure S2C), as previously described (Homem and Peifer,

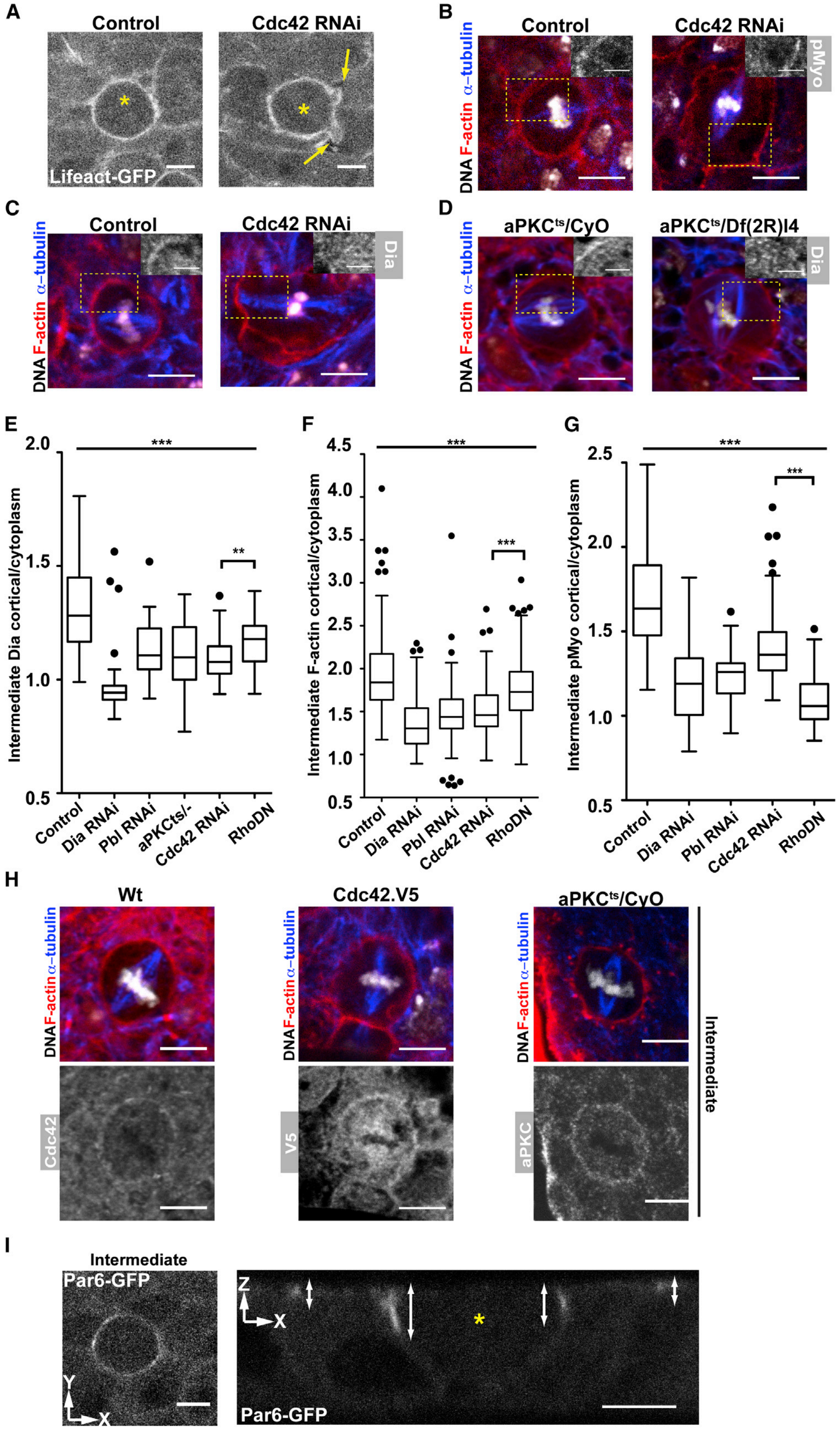

(legend on next page)

2008), but was largely absent from the basolateral cortex (Figure S2E). Then, upon entry into mitosis, Dia accumulated at lateral membranes (Figure 3E) before moving to the spindle midzone, where it is required for nucleation of the cytokinesis ring. By contrast, in Pbl RNAi flies, Dia was lost from the metaphase cell cortex and reduced at interphase AJs (Figures 3E, 4E, and S2E). In addition, there was a marked reduction in the levels of phosphorylated Myosin-II (pMyo) in the metaphase cortex of flies expressing Pbl dsRNA (Figures 3D, 4G, and S2D). These data identify Pbl as a master regulator of cortical assembly in fly cells, as was previously shown for Ect2 in human cells (Matthews et al., 2012).

To follow the pathway downstream of the RhoGEF Pbl, we used a dominant-negative Rho1 protein to test the effects of Rho1 inhibition in this tissue (since expression of Rho1 RNAi proved to be highly toxic). Rho1DN was sufficient to induce loss of cortical pMyosin-II (Figures 3D, 4G, and S2D). Further, as expected (O'Keefe et al., 2001; Prokopenko et al., 2000), cells expressing this dominant-negative Rho1 transgene failed to complete cytokinesis, as evidenced by the presence of metaphase cells with larger mitotic spindles and increased DNA content (Figures S2D and S2E). Given these phenotypes, the comparatively modest reduction in cortical levels of Dia and F-actin observed in Rho1DN cells was surprising (Figures 3E, 4E, and 4F). This suggested the possibility that there might be downstream effectors of Pbl other than Rho1 that are substrates of its broad guanine exchange factor (GEF) activity (van Impel et al., 2009).

Cdc42/aPKC/Par6 Control Cortical Dia Localization in Mitosis

In biochemical assays, Pbl/Ect2 has been shown to catalyze GDP-GTP exchange on RhoA, Rac, and Cdc42 (Tatsumoto et al., 1999). While Rac is not known to have an important mitotic function, in human cells levels of GTP-Cdc42 have been reported to peak in metaphase in a Pbl/Ect2-dependent manner (Oceguera-Yanez et al., 2005). Moreover, a wide range of mitotic defects have been attributed to loss of Cdc42 (Narumiya and Yasuda, 2006). This led us to test whether Cdc42 might contribute to mitotic cortex assembly downstream of Pbl.

To do so, we first used UAS-Lifeact::GFP to image the mitotic cortex in epithelial tissue expressing Cdc42 dsRNA. Although Cdc42 silencing was not sufficient to abrogate assembly of a mitotic F-actin cortex, Cdc42 RNAi cells exhibited profound defects in cortical stability. In these cells, the F-actin cortex underwent continuous blebbing (Figure 4A; Figure S3A)—something never seen in the control. Similarly, cortical defects including blebbing were seen in fixed preparations of Cdc42 RNAi cells (Figures 4B and 4C) and in small null mutant *cdc42*^{3} clones visualized live using UAS-Tub::GFP (Figure S3B). Furthermore, cortical Dia was found to be largely absent from the lateral cortex of metaphase cells expressing Cdc42 dsRNA (Figures 4C and 4E), implying a role for Cdc42, like Pbl, in Dia localization, even though changes in the levels of pMyosin-II in Cdc42 RNAi cells (Figures 4B and 4G) were much less marked than those seen in Rho1DN expressing tissues (Figures 3D and 4G). Thus, Rho1 and Cdc42 appear to have complementary activities: Dia localization depends on Cdc42, whereas Myosin-II activation depends on Rho1.

Since Cdc42 functions together with its binding partners aPKC and Par6 in generating cortical F-actin-based structures in interphase epithelial cells of the fly notum (Georgiou and Baum, 2010; Georgiou et al., 2008), it was important to determine whether aPKC and Par6 also contribute to the assembly of a stable mitotic cortex. We took advantage of a recently reported temperature-sensitive allele of *aPKC* (*aPKC*^{ts}) (Guilgur et al., 2012) to perturb the function of aPKC in intact epithelia. At restrictive temperatures, mitotic *aPKC*^{ts} mutant cells (*aPKC*^{ts}*/Df(2R)l4*) resembled Cdc42 RNAi cells in exhibiting large bleb-like deformations (Figure S3C), a severely disrupted metaphase cortex (Figure 4D), and reduced levels of cortical Dia (Figures 4D and 4E), relative to the control (*aPKC*^{ts}*/CyO*). Further, using the *Gal80ts* line to limit *pnr-Gal4*-mediated expression of dsRNAs targeting aPKC and Par6 to specific pupal stages, we observed similar defects in cortical F-actin organization to those described in Cdc42 RNAi cells (Figure S3C). Taken together, these data suggest that Cdc42 functions together with the polarity proteins aPKC and Par6 to control the assembly of a stable Dia-dependent mitotic actin cortex.

Recent studies have implicated an important role for epithelial polarity factors, including Cdc42, Par6, and aPKC (Durgan et al., 2011; Guilgur et al., 2012; Hao et al., 2010; Jaffe et al., 2008), in spindle orientation during symmetric cell divisions (Bergstralh

Figure 4. Cdc42 and aPKC Are Required for Cortical Accumulation of Dia

(A) Intermediate section in plane of epithelium (xy) of metaphase cells in control and Cdc42 RNAi epithelia expressing Lifeact::GFP (yellow arrow indicates cell blebs).

(B and C) Metaphase cells from control flies expressing *pnr*-Gal4 and Cdc42 RNAi stained for (B) F-actin (red), DNA (white), α-tubulin (blue), and pMyosin-II (Inset, white), or (C) Dia (Inset, white). Scale bars, 5 μm.

(D) Control and mutant aPKC^{ts} (heterozygous aPKC^{ts}/CyO and hemizygous aPKC^{ts}/Df(2R)l4, respectively) stained for F-actin (red), DNA (white), α-tubulin (blue), and Dia (Inset, white). Scale bars, 5 μm.

(E) Box plot showing intermediate cortical/cytoplasmic Dia staining in metaphase cells from control tissue, and tissue expressing dsRNAs for Dia, Pbl, or Cdc42, Rho1.N19 (DN), and in an aPKC^{ts} mutant. A nonparametrical Mann-Whitney test was used to confirm significance (**$p < 0.05$) between Cdc42 RNAi and Rho1.N19 expressing nota.

(F and G) Box plot showing cortical/cytoplasmic (F) F-actin (Phalloidin staining) and (G) phospho-Myosin-II ratios for metaphase cells from control, Dia, Pbl, Cdc42 RNAi and Rho1.N19 nota. Statistically significant differences were observed (***$p < 0.001$) between Cdc42 RNAi and RhoDN expressing nota (non-parametrical Mann-Whitney test). A one-way ANOVA test was used to confirm significance of difference (***$p < 0.001$) between control and treated nota in all box plot graphs ($n \geq 30$ cells from at least 3 different pupae).

(H) Metaphase cells shown in plane of epithelium (xy) in control, Cdc42.V5 expressing and aPKC^{ts}/CyO mutant tissue stained for F-actin (red), DNA (white), α-tubulin (blue), and Cdc42, V5, and aPKC (white), respectively.

(I) Live metaphase cell expressing Par6::GFP shown in plane of epithelium (left, xy) and in cross-section (right, xz). White double arrow marks the Par6::GFP profile. Yellow asterisk marks metaphase cell. Scale bars, 5 μm.

See also Figure S3.

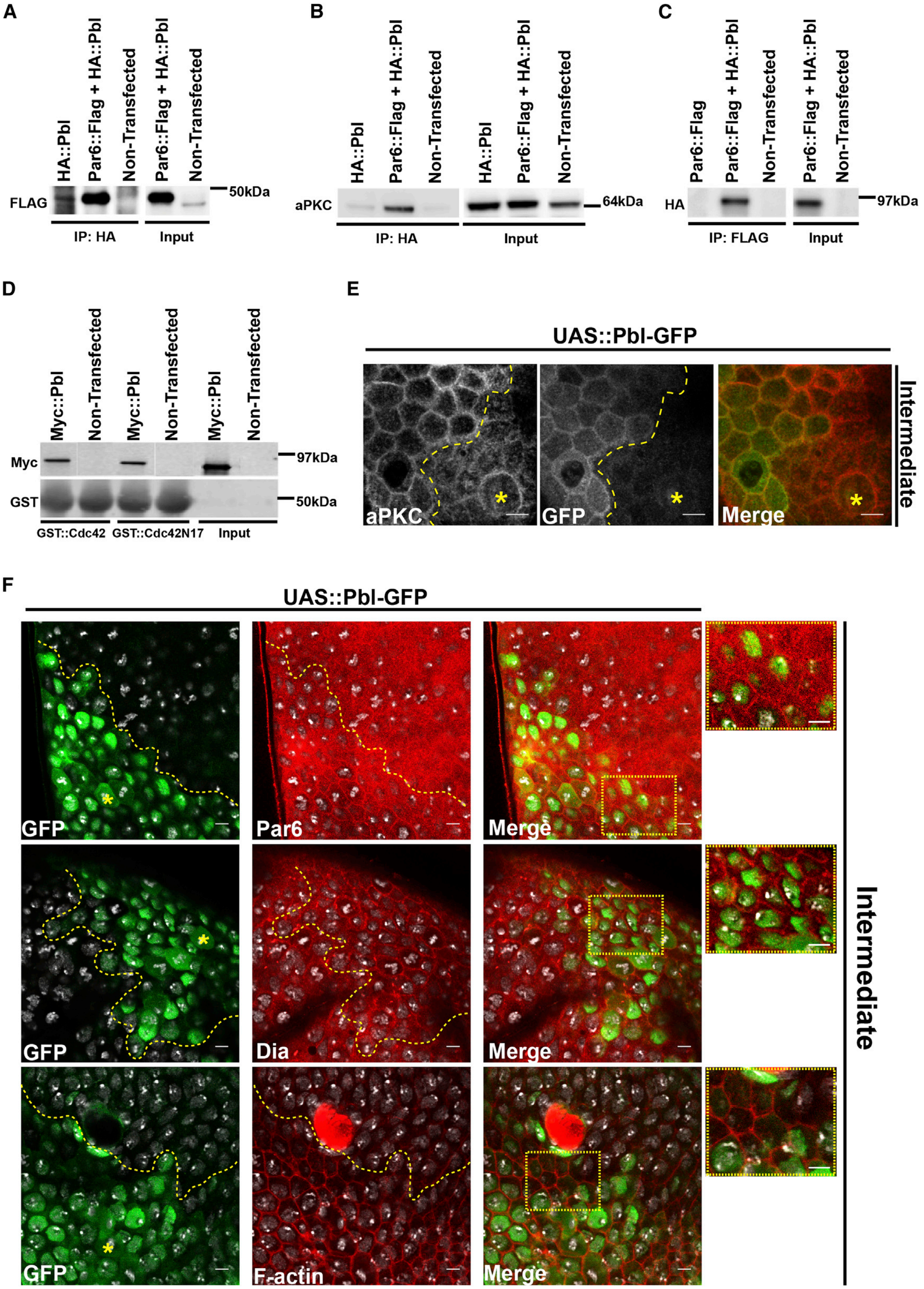

Figure 5. Pbl Interacts with Par6 and Cdc42

Complexes were immunoprecipitated (IP) from S2 lysates transfected with control (salmon sperm DNA), HA::Pbl, or HA::Pbl and Par6::FLAG constructs using an anti-HA or anti-Flag antibody.

(A–C) Western blot analyses were conducted with anti-Flag (A), anti-aPKC (B), or anti-HA (C) antibodies.

(legend continued on next page)

et al., 2013; Durgan et al., 2011; Guilgur et al., 2012; Hao et al., 2010; Jaffe et al., 2008; Nakajima et al., 2013). However, while modest spindle orientation defects have been reported in *Drosophila* wing discs lacking aPKC (Guilgur et al., 2012), spindle orientation in the follicular epithelium appears to rely on the lateral factor Discs-large instead of aPKC (Bergstralh et al., 2013). This led us to analyze spindle orientation in Cdc42 and Dia RNAi conditions and in *aPKC*^{ts} mutant nota. In each case, centrosome separation appeared unaffected, and cells divided in the plane of the epithelium. The only defects we observed were minor changes in spindle movements in Dia RNAi cells (data not shown). Together, these data suggest that, in cells of the notum, Cdc42/aPKC/Par6 aid the assembly of the actomyosin cortex, rather than formation or orientation of the mitotic spindle.

Mitotic Entry Is Accompanied by a Shift in Cdc42/aPKC/Par6 Localization

In interphase *Drosophila* epithelial cells, Cdc42/aPKC/Par6 proteins are confined to a well-defined apical domain, where they perform important roles in the regulation of junctional organization and cell polarity (St Johnston and Ahringer, 2010). How then can Cdc42, aPKC, and Par6 contribute to the mitotic redistribution of Dia and to the formation of a relatively uniform, rounded metaphase cortex? To address this question, we used antibodies and GFP-fusion proteins to determine the localization of these polarity proteins in mitotic cells in fixed tissues. This revealed an apical, junctional pool of Cdc42 and aPKC in interphase cells (Figures S4A and S4C), as previously described (Harris and Tepass, 2008). However, at mitotic entry, both Cdc42 and aPKC extended their domain of localization along the lateral cortex (Figure 4H). The specificity of this staining was confirmed using Cdc42 RNAi (Figures S4A and S4B) and by comparing *aPKC*^{ts}*/CyO* and *aPKC*^{ts} (*aPKC*^{ts}*/Df(2R)l4*) cells at the restrictive temperature (Figures S4C and S4D [Guilgur et al., 2012]). Since we were unable to observe robust staining for Par6 using available antibodies, we imaged expression of a functional Par6-GFP fusion protein instead (Wirtz-Peitz et al., 2008). As expected, this revealed a basal shift in the localization of Par6-GFP along the lateral cortex in cells entering mitosis (Figure 4I), a process that was reversed at mitotic exit (Figure S4E). This shift in the localization of the polarity proteins Cdc42, aPKC, and Par6 during mitotic entry could therefore contribute to the generation of a stable rounded cortex.

Ect2/Pbl Can Act through Cdc42 and Dia to Redirect Cortical Actin Assembly

Ect2 can activate PKCζ and Cdc42 via direct binding to the Par6/Par3/PKCζ complex to regulate the establishment of epithelial cell polarity (Liu et al., 2004, 2006). Because of this, we wondered whether Pbl might associate with the homologous polarity proteins in fly cells. To look for a biochemical interaction, we carried out co-immunoprecipitation assays using fly S2 cell extracts transfected with Par-6 fused with Flag epitope (Par6::Flag) and Pbl fused with HA epitope (Pbl::HA). In this assay, Pbl::HA and Par6::FLAG associated with one another in experiments carried out in either direction (Figures 5A and 5C). The interaction was confirmed by pulling down Myc::Pbl transfected cells with Par6::GST (glutathione S-transferase) beads (data not shown). Endogenous aPKC protein only came down in cells expressing both Pbl::HA I and Par6::Flag (Figure 5B). However, Cdc42WT::GST was found associated with Pbl in pull-downs (Figure 5D), as was Cdc42.N17::GST, as expected for a substrate of a GEF.

These data support the idea of there being a physical association of Pbl with Par6/aPKC/Cdc42 complex at the onset of mitosis. If the activation and relocalization of Pbl to the cytoplasm of mitotic cells were sufficient to repolarize the cell and to induce the lateral recruitment of active Dia, it might be possible to mimic the formation of the mitotic cortex through the simple overexpression of Pbl. To test whether this is the case, we used Gal80ts in combination with *pnr*-Gal4 to ectopically express Pbl::GFP in isolated clusters of cells in the fly notum. Remarkably, this drove the lateral displacement of aPKC in interphase (Figure 5E) in a manner that was comparable to that seen in mitosis (compare cells in *Pnr* domain with mitotic cell marked with yellow asterisk). Moreover, like their mitotic counterparts, these Pbl::GFP positive cells exhibited an increase of cortical Dia, Par6, and F-actin (Figure 5F).

Similarly, if assembly of the F-actin cortex downstream of Pbl depends on Cdc42 and Dia, the activation of either protein should also promote the assembly of a mitotic-like cortex. Since the expression of constitutively active forms of Cdc42 and Dia (Cdc42.V12 and Dia^{CA}) proved to be highly toxic, to carry out this test we used *Tub-Gal80ts Neu-GMA* to limit expression to precise periods of SOP development. Strikingly, the expression of either Cdc42.V12 or Dia^{CA} was sufficient to drive the assembly of a rounded, F-actin-rich cortex in interphase cells (Figure 6A) in the absence of significant amounts of cytoplasmic Pbl/Ect2. Furthermore, when these SOP cells were followed through their first round of cell division, they exhibited profound cell shape instabilities and blebbing (Figure 6B). While the phenotypes were similar in the two cases, the effects of Dia^{CA} were reproducibly stronger, leading to a thicker actin cortex, blebbing, and defects in spindle orientation (Figure S5B). In certain instances these defects were so extreme as to cause blebs to be pinched off and lost (Figure S5D). Interestingly, the expression of constitutively active forms of Cdc42 and Dia was also sufficient to induce the formation of an actin rich cortex at the apical surface of cells (Figures S5A and S5C). Since actomyosin was cleared from the apical region of control mitotic cells, these data imply that local Pbl activation provides the positional information required to position active Cdc42 and Dia, which then drive local cortical assembly.

(D) Equal amounts of S2 cell lysates transfected with Myc::Pbl were subjected to pull-down assays with GST::Cdc42WT or GST::Cdc42.N17. Pbl was detected by immunoblotting with anti-Myc antibody. GST staining is shown at the bottom to visualize recombinant Cdc42 proteins.

(E) Intermediate top view of fly nota expressing Pbl-GFP in subsets of cells at the limits of the *pnr* domain stained for GFP (green) and aPKC (red).

(F) Pbl-GFP (green), Par6, Dia, and Phalloidin (red) staining of three fly nota. Yellow asterisk marks mitotic cells in the *pnr* domain. Yellow dashed square: inset of *pnr* domain frontier.

See also Figure S4.

Cell Press

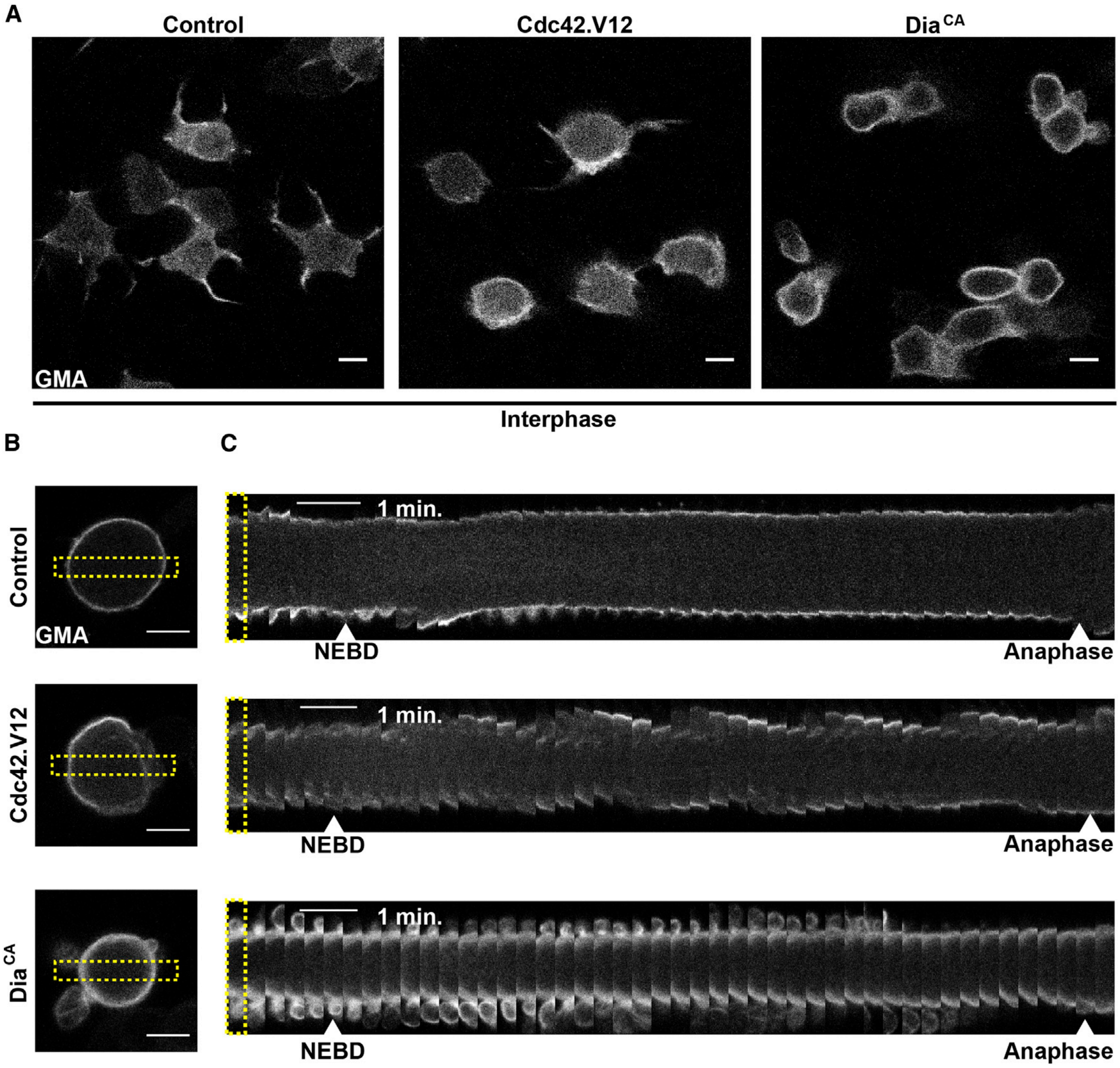

Figure 6. Cdc42.V12 or Dia^{CA} Expression Is Sufficient to Induce Cell Rounding in Interphase

(A) Intermediate level view in plane of epithelium of control, Cdc42.V12, and Dia^{CA} expressing SOP cells marked with GMA::GFP to label F-actin in interphase.
(B) Similar views of control, Cdc42.V12, and Dia^{CA} expressing SOP cells marked with GMA::GFP in metaphase. Scale bars, 5 μm.
(C) Kymograph of cell shown in (B). Yellow dashed rectangle marks the region used for the generation of kymograph. Scale bars, 1 min.
See also Figure S5.

DISCUSSION

Studies in cell culture systems have shown that the release of Ect2/Pbl from the nucleus at mitotic entry (Matthews et al., 2012) activates Rho (Maddox and Burridge, 2003) to drive F-actin (Cramer and Mitchison, 1997) and Myosin-II dependent mitotic rounding. Here, we show that cytoplasmic Pbl/Ect2 plays a similar role in the assembly of a metaphase actomyosin cortex in epithelial cells in the developing fly. In human cells, this change in cell shape appears to be triggered by rising levels of nuclear Cdk1/CyclinB, which leads to the export of activated Ect2/Pbl (Matthews et al., 2012). Although threonine T341, a key site of Cdk1/CyclinB-mediated Ect2 phosphorylation, is not conserved in Pbl (Hara et al., 2006), the C-terminal is highly conserved and includes T814, another conserved site of Cdk1/CyclinB-mediated phosphorylation (Niiya et al., 2006; Su et al., 2011). Thus, these data suggest the possibility that Pbl/Ect2 is regulated in a similar fashion across animal species, enabling it to perform a conserved function in driving changes in mitotic cell shape.

The current study identifies Dia as the critical regulator of actin nucleation downstream of Pbl/Ect2 required for assembly of the mitotic cortex. Moreover, using live imaging we have been able to observe a rapid shift in cortical organization at mitotic entry that depends on Dia, but not Arp3. While studies in human cells have yet to identify the actin substrate upon which Myosin-II acts to drive mitotic rounding, Dia homologs likely play a key role (Bovellan et al., 2014). Given the well-established role for Dia in cytokinesis (O'Keefe et al., 2001; Prokopenko et al., 1999), its identification as the critical actin nucleator required for the generation of the metaphase cortex strengthens the idea that it is similar in

composition to the circular contractile actomyosin ring used to drive cell division (Matthews et al., 2012). Importantly, however, our work identifies a key difference between the pathways involved in these two processes: while Rho1 is absolutely required for the localization of Dia and Myosin-II and the assembly of the actomyosin ring at cytokinesis, the metaphase cortex relies on the collaboration of Rho and Cdc42. In metaphase, Cdc42, Par6, and aPKC play the dominant role in the control of Dia localization, while Rho appears more important for the activation of Myosin-II. (Note that we observed little evidence of Cdc42 or aPKC accumulating within the cytokinetic ring.) Since Pbl/Ect2 is required for both actin nucleation and Myosin-II activation during metaphase, it is likely that this requires the downstream activation of both Rho and the Cdc42/aPKC/Par6 complex.

While a role for Cdc42 in the localization of Dia during mitotic rounding might seem surprising, previous studies have suggested roles for the mitotic activation of Cdc42 (Hutterer et al., 2004) in mitotic actin assembly (Zhu et al., 2011). Moreover, in yeast, the localization and activation of formins is dependent both on the Rho GTPase Cdc42 and the protein kinase C Pkc1p (Dong et al., 2003). These data suggest a conserved role for aPKC, Rho, and Cdc42 in the control of actin nucleators during mitosis. Interestingly, while a large number of studies have implicated Cdc42 in spindle orientation (Gotta et al., 2001; Jaffe et al., 2008; Mitsushima et al., 2009), in line with a recent study in the fly egg chamber (Bergstralh et al., 2013), we find no evidence for a role of Cdc42, aPKC, or Par6 in the regulation of spindle alignment in the fly notum. However, since spindle defects can arise from defects in mitotic cell shape (Lancaster et al., 2013), these previously observed phenotypes may be an indirect consequence of the role of polarity proteins in the assembly of a rigid actomyosin cortex.

If Pbl/Ect2 functions to coordinate the activities of Rho and Cdc42, how might the switch in Pbl/Ect2 activity from metaphase (Cdc42 and Rho) to anaphase (Rho) be accomplished? One possibility is that this results from changes in the status of Pbl/Ect2 phosphorylation sites that accompany mitotic progression and change the specificity of the Pbl/Ect2 GEF activity toward different substrates, e.g., like the switch in Pbl activity from Rho to Rac-dependent cell motility during mesoderm migration in flies (van Impel et al., 2009). In addition, levels of active Rho family GTPases during mitotic progression will be affected by the activity and location of counteracting RhoGAPs (Oceguera-Yanez et al., 2005), many of which are known to display different activities toward different Rho family GTPases (Touré et al., 1998). Finally, this switch in behavior could rely on the function stage or location specific accessory proteins, such as BAR domain proteins (Ren et al., 2006).

Although the pathway leading from Pbl/Ect2 to Rho and Myosin-II was first discovered in human cells in culture (Cramer and Mitchison, 1997; Maddox and Burridge, 2003; Matthews et al., 2012), rounding under these conditions does not require an intact F-actin cytoskeleton (Lancaster et al., 2013). In an epithelium, by contrast, significant actomyosin-based forces are required to drive rounding, since the dividing cells have to make space within the tissue in which to build a mitotic spindle (Lancaster et al., 2013; Luxenburg et al., 2011; Nakajima et al., 2013). In the fly notum, it is clear that the establishment of a relatively uniform contractile actomyosin cortex downstream of Pbl/Ect2 plays a critical role in ensuring that cells assume a rigid, spherical state. Thus, cells lacking an F-actin cortex, as the result of Pbl or Dia RNAi, have a variable shape that often departs markedly from the robust, spherical form of control cells. Conversely, cells expressing activated forms of either Dia or Cdc42 exhibit uncontrolled cortical blebbing, showing the importance of fine-tuning cortical forces.

Interestingly, in many systems Cdc42 functions as a master regulator of the polarized localization of different populations of F-actin (Johnson, 1999). More specifically, during interphase in epithelial cells within the developing fly, apically localized Cdc42 has been proposed to function together with aPKC and Par6 to control a large set of distinct F-actin-based structures. These include apically localized Dia at cell-cell junctions (Warner and Longmore, 2009), the activation of Wasp-dependent AJ endocytosis (Georgiou et al., 2008), and, through interactions with Baz, Tiam1, and Rac, the activation of basal Arp2/3-based protrusions (Georgiou and Baum, 2010). Therefore, one of the primary effects of the observed shift in the localization of Cdc42/Par6/aPKC upon entry into mitosis may be the depolarization of this spatially differentiated F-actin cytoskeleton. As these proteins bleed along the lateral cell membrane, they appear to recruit Dia, enabling the nucleation of a relatively isotropic actomyosin cortex. In this way, passage into and out of mitosis involves an unexpectedly tight coordination of cell polarity and actin cytoskeletal remodeling.

EXPERIMENTAL PROCEDURES

Fly Stocks

The following stocks were used: *pnr-GAL4* (Bloomington:3039), *aPKC*^{ts} (Guilgur et al., 2012), *cdc42*^{3} (Fehon et al., 1997), *tub-GAL80*^{ts} (Bloomington:7108), *UAS-Dia*^{CA} (Bloomington:27616), *UAS-Cdc42*^{V12} (Bloomington:6287), *neu-GMA* (Edwards et al., 1997; Kunda et al., 2012), *neu-GAL4* (Bellaïche et al., 2001), *Lifeact*::*GFP* (Hatan et al., 2011), *sqh*^{Ax3}*; Sqh*::*GFP* (Monier et al., 2010), *Cdc42*^{V5} (Fletcher et al., 2012), *UAS-Tub*::*RFP* (McGill et al., 2009), and *UAS-Pbl*::*GFP* (Somers and Saint, 2003). Vienna Drosophila Resource Center (VDRC) RNAi lines were used to silence the expression of the genes *aPKC*, *arp3*, *diaphanous*, *pebble*, and *par-6*, and a NIG-Fly library line was used for *cdc42*. For Flybase ID, see Supplemental Experimental Procedures.

Dissections and Live Imaging

For time-lapse acquisition experiments, animals expressing the appropriate reporter were imaged through a window in the pupal case under a drop of injection oil via confocal microscopy at room temperature (Georgiou and Baum, 2010). Time-lapse movies were acquired using an upright Leica SPE confocal microscope. In vivo live imaging of pupal nota was performed at 14–16 hr after pupariation (AP) and labeled using a variety of markers (see Supplemental Experimental Procedures). RNAi-induced gene silencing was accomplished by using the *pnr*-Gal4 driver to express Gal4-responsive hairpin dsRNAs in transgenic flies (Mummery-Widmer et al., 2009). For fixed preparations, nota from 14 to 16 hr AP were promptly fixed in 4% formaldehyde for 20 min at room temperature, before being permeabilized in PBS containing 0.1% Triton X-100. Subsequently, nota were incubated in a blocking solution composed of 5% BSA and 3% fetal bovine serum (FBS) (in PBS), preceding antibody incubation (see Supplemental Experimental Procedures). Imaging was performed using an inverted Leica SP5 confocal microscope.

Cell Culture and Immunoprecipitation

S2 cells (DGRC) were cultured in Schneider's medium (GIBCO) supplemented with 10% heat inactivated FBS (GIBCO) and 1% penicillin/streptomycin (GIBCO) at 25°C. Transfection was performed using Effectene Transfection

Reagent (QIAGEN). A total of 1 μg of plasmid DNA was used per well. Cell lysates were incubated with 5 μg of antibody (see Supplemental Experimental Procedures) and immunoprecipitated with Protein A/G magnetic beads (Pierce, Thermo Scientific). For western blot analysis, gels were blotted to a polyvinylidene difluoride membrane (see Supplemental Experimental Procedures). Protein signals were detected by enhanced chemiluminescence (Biological Industries).

In Vitro Binding Assay

For the glutathione S-transferase pull-down assays, 400 μl of cell lysate was incubated with an excess of GST fusion proteins immobilized on glutathione Sepharose beads (see Supplemental Experimental Procedures). Proteins were resolved by SDS-PAGE and detected using the LICOR Odyssey scanner (Li-Cor Bioscience).

Image Processing and Analysis

The images presented were processed with Fiji (Schindelin et al., 2012) and Adobe Illustrator CS (Adobe Systems). Intensity profiles at the cortex of cells were quantified in a single confocal z medial slice using ImageJ. A line (of 10 pixels width) was outlined around the cortex of cells to measure gray mean intensity (analyze > measure > mean pixel intensity). The same procedure was performed for a line of equal length inside the cell (avoiding the DNA). The cortical/cytoplasmic ratio was given by the ratio of the mean pixel intensity on the cortex of cells divided by the mean gray intensity inside the cell. Statistics were calculated on the basis of a minimum of 30 cells in at least 3 different pupae.

SUPPLEMENTAL INFORMATION

Supplemental Information includes Supplemental Experimental Procedures and five figures and can be found with this article online at http://dx.doi.org/10.1016/j.devcel.2015.01.012.

AUTHOR CONTRIBUTIONS

A.R. designed and carried out all the experiments described in the paper under the guidance of B.B., except for the biochemical experiments, which were carried out in collaboration with E.V. F.P. helped to plan these experiments. The paper was written by A.R. and B.B.

ACKNOWLEDGMENTS

We are grateful to Barry Thompson, Rui Martinho, Arno Müller, and Steve Wasserman for fly lines and antibodies. A.R. was supported by the Fundação para a Ciência e Tecnologia grant SFRH/BD/33543/2008. B.B.'s lab was supported by a Cancer Research UK grant (9786) and a BBSRC grant (BB/K009001/1), and F.P. and E.V. were supported by the MRC.

Received: March 1, 2014
Revised: October 6, 2014
Accepted: January 14, 2015
Published: February 19, 2015

REFERENCES

Bellaïche, Y., Gho, M., Kaltschmidt, J.A., Brand, A.H., and Schweisguth, F. (2001). Frizzled regulates localization of cell-fate determinants and mitotic spindle rotation during asymmetric cell division. Nat. Cell Biol. *3*, 50–57.

Bergstralh, D.T., Lovegrove, H.E., and St Johnston, D. (2013). Discs large links spindle orientation to apical-basal polarity in *Drosophila* epithelia. Curr. Biol. *23*, 1707–1712.

Bosveld, F., Bonnet, I., Guirao, B., Tlili, S., Wang, Z., Petitalot, A., Marchand, R., Bardet, P.L., Marcq, P., Graner, F., and Bellaïche, Y. (2012). Mechanical control of morphogenesis by Fat/Dachsous/Four-jointed planar cell polarity pathway. Science *336*, 724–727.

Bovellan, M., Romeo, Y., Biro, M., Boden, A., Chugh, P., Yonis, A., Vaghela, M., Fritzsche, M., Moulding, D., Thorogate, R., et al. (2014). Cellular control of cortical actin nucleation. Curr. Biol. *24*, 1628–1635.

Carreno, S., Kouranti, I., Glusman, E.S., Fuller, M.T., Echard, A., and Payre, F. (2008). Moesin and its activating kinase Slik are required for cortical stability and microtubule organization in mitotic cells. J. Cell Biol. *180*, 739–746.

Castrillon, D.H., and Wasserman, S.A. (1994). Diaphanous is required for cytokinesis in *Drosophila* and shares domains of similarity with the products of the limb deformity gene. Development *120*, 3367–3377.

Cramer, L.P., and Mitchison, T.J. (1997). Investigation of the mechanism of retraction of the cell margin and rearward flow of nodules during mitotic cell rounding. Mol. Biol. Cell *8*, 109–119.

Dao, V.T., Dupuy, A.G., Gavet, O., Caron, E., and de Gunzburg, J. (2009). Dynamic changes in Rap1 activity are required for cell retraction and spreading during mitosis. J. Cell Sci. *122*, 2996–3004.

Dong, Y., Pruyne, D., and Bretscher, A. (2003). Formin-dependent actin assembly is regulated by distinct modes of Rho signaling in yeast. J. Cell Biol. *161*, 1081–1092.

Durgan, J., Kaji, N., Jin, D., and Hall, A. (2011). Par6B and atypical PKC regulate mitotic spindle orientation during epithelial morphogenesis. J. Biol. Chem. *286*, 12461–12474.

Edwards, K.A., Demsky, M., Montague, R.A., Weymouth, N., and Kiehart, D.P. (1997). GFP-moesin illuminates actin cytoskeleton dynamics in living tissue and demonstrates cell shape changes during morphogenesis in *Drosophila*. Dev. Biol. *191*, 103–117.

Fededa, J.P., and Gerlich, D.W. (2012). Molecular control of animal cell cytokinesis. Nat. Cell Biol. *14*, 440–447.

Fehon, R.G., Oren, T., LaJeunesse, D.R., Melby, T.E., and McCartney, B.M. (1997). Isolation of mutations in the *Drosophila* homologues of the human Neurofibromatosis 2 and yeast CDC42 genes using a simple and efficient reverse-genetic method. Genetics *146*, 245–252.

Fink, J., Carpi, N., Betz, T., Bétard, A., Chebah, M., Azioune, A., Bornens, M., Sykes, C., Fetler, L., Cuvelier, D., and Piel, M. (2011). External forces control mitotic spindle positioning. Nat. Cell Biol. *13*, 771–778.

Fletcher, G.C., Lucas, E.P., Brain, R., Tournier, A., and Thompson, B.J. (2012). Positive feedback and mutual antagonism combine to polarize Crumbs in the *Drosophila* follicle cell epithelium. Curr. Biol. *22*, 1116–1122.

Founounou, N., Loyer, N., and Le Borgne, R. (2013). Septins regulate the contractility of the actomyosin ring to enable adherens junction remodeling during cytokinesis of epithelial cells. Dev. Cell *24*, 242–255.

Georgiou, M., and Baum, B. (2010). Polarity proteins and Rho GTPases cooperate to spatially organise epithelial actin-based protrusions. J. Cell Sci. *123*, 1089–1098.

Georgiou, M., Marinari, E., Burden, J., and Baum, B. (2008). Cdc42, Par6, and aPKC regulate Arp2/3-mediated endocytosis to control local adherens junction stability. Curr. Biol. *18*, 1631–1638.

Gotta, M., Abraham, M.C., and Ahringer, J. (2001). CDC-42 controls early cell polarity and spindle orientation in *C. elegans*. Curr. Biol. *11*, 482–488.

Grosshans, J., Wenzl, C., Herz, H.M., Bartoszewski, S., Schnorrer, F., Vogt, N., Schwarz, H., and Müller, H.A. (2005). RhoGEF2 and the formin Dia control the formation of the furrow canal by directed actin assembly during *Drosophila* cellularisation. Development *132*, 1009–1020.

Guilgur, L.G., Prudêncio, P., Ferreira, T., Pimenta-Marques, A.R., and Martinho, R.G. (2012). *Drosophila* aPKC is required for mitotic spindle orientation during symmetric division of epithelial cells. Development *139*, 503–513.

Guillot, C., and Lecuit, T. (2013). Adhesion disengagement uncouples intrinsic and extrinsic forces to drive cytokinesis in epithelial tissues. Dev. Cell *24*, 227–241.

Hao, Y., Du, Q., Chen, X., Zheng, Z., Balsbaugh, J.L., Maitra, S., Shabanowitz, J., Hunt, D.F., and Macara, I.G. (2010). Par3 controls epithelial spindle orientation by aPKC-mediated phosphorylation of apical Pins. Curr. Biol. *20*, 1809–1818.

Hara, T., Abe, M., Inoue, H., Yu, L.R., Veenstra, T.D., Kang, Y.H., Lee, K.S., and Miki, T. (2006). Cytokinesis regulator ECT2 changes its conformation through phosphorylation at Thr-341 in G2/M phase. Oncogene *25*, 566–578.

Harris, K.P., and Tepass, U. (2008). Cdc42 and Par proteins stabilize dynamic adherens junctions in the *Drosophila* neuroectoderm through regulation of apical endocytosis. J. Cell Biol. *183*, 1129–1143.

Hatan, M., Shinder, V., Israeli, D., Schnorrer, F., and Volk, T. (2011). The *Drosophila* blood brain barrier is maintained by GPCR-dependent dynamic actin structures. J. Cell Biol. *192*, 307–319.

Herszterg, S., Leibfried, A., Bosveld, F., Martin, C., and Bellaiche, Y. (2013). Interplay between the dividing cell and its neighbors regulates adherens junction formation during cytokinesis in epithelial tissue. Dev. Cell *24*, 256–270.

Homem, C.C., and Peifer, M. (2008). Diaphanous regulates myosin and adherens junctions to control cell contractility and protrusive behavior during morphogenesis. Development *135*, 1005–1018.

Hutterer, A., Betschinger, J., Petronczki, M., and Knoblich, J.A. (2004). Sequential roles of Cdc42, Par-6, aPKC, and Lgl in the establishment of epithelial polarity during *Drosophila* embryogenesis. Dev. Cell *6*, 845–854.

Jaffe, A.B., Kaji, N., Durgan, J., and Hall, A. (2008). Cdc42 controls spindle orientation to position the apical surface during epithelial morphogenesis. J. Cell Biol. *183*, 625–633.

Johnson, D.I. (1999). Cdc42: An essential Rho-type GTPase controlling eukaryotic cell polarity. Microbiol. Mol. Biol. Rev. *63*, 54–105.

Kondo, T., and Hayashi, S. (2013). Mitotic cell rounding accelerates epithelial invagination. Nature *494*, 125–129.

Kunda, P., and Baum, B. (2009). The actin cytoskeleton in spindle assembly and positioning. Trends Cell Biol. *19*, 174–179.

Kunda, P., Pelling, A.E., Liu, T., and Baum, B. (2008). Moesin controls cortical rigidity, cell rounding, and spindle morphogenesis during mitosis. Curr. Biol. *18*, 91–101.

Kunda, P., Rodrigues, N.T., Moeendarbary, E., Liu, T., Ivetic, A., Charras, G., and Baum, B. (2012). PP1-mediated moesin dephosphorylation couples polar relaxation to mitotic exit. Curr. Biol. *22*, 231–236.

Lancaster, O.M., Le Berre, M., Dimitracopoulos, A., Bonazzi, D., Zlotek-Zlotkiewicz, E., Picone, R., Duke, T., Piel, M., and Baum, B. (2013). Mitotic rounding alters cell geometry to ensure efficient bipolar spindle formation. Dev. Cell *25*, 270–283.

Liu, X.F., Ishida, H., Raziuddin, R., and Miki, T. (2004). Nucleotide exchange factor ECT2 interacts with the polarity protein complex Par6/Par3/protein kinase Czeta (PKCzeta) and regulates PKCzeta activity. Mol. Cell. Biol. *24*, 6665–6675.

Liu, X.F., Ohno, S., and Miki, T. (2006). Nucleotide exchange factor ECT2 regulates epithelial cell polarity. Cell. Signal. *18*, 1604–1615.

Lu, B., Roegiers, F., Jan, L.Y., and Jan, Y.N. (2001). Adherens junctions inhibit asymmetric division in the *Drosophila* epithelium. Nature *409*, 522–525.

Luxenburg, C., Pasolli, H.A., Williams, S.E., and Fuchs, E. (2011). Developmental roles for Srf, cortical cytoskeleton and cell shape in epidermal spindle orientation. Nat. Cell Biol. *13*, 203–214.

Maddox, A.S., and Burridge, K. (2003). RhoA is required for cortical retraction and rigidity during mitotic cell rounding. J. Cell Biol. *160*, 255–265.

Marinari, E., Mehonic, A., Curran, S., Gale, J., Duke, T., and Baum, B. (2012). Live-cell delamination counterbalances epithelial growth to limit tissue overcrowding. Nature *484*, 542–545.

Matthews, H.K., Delabre, U., Rohn, J.L., Guck, J., Kunda, P., and Baum, B. (2012). Changes in Ect2 localization couple actomyosin-dependent cell shape changes to mitotic progression. Dev. Cell *23*, 371–383.

McGill, M.A., McKinley, R.F., and Harris, T.J. (2009). Independent cadherin-catenin and Bazooka clusters interact to assemble adherens junctions. J. Cell Biol. *185*, 787–796.

Mitsushima, M., Toyoshima, F., and Nishida, E. (2009). Dual role of Cdc42 in spindle orientation control of adherent cells. Mol. Cell. Biol. *29*, 2816–2827.

Monier, B., Pelissier-Monier, A., Brand, A.H., and Sanson, B. (2010). An actomyosin-based barrier inhibits cell mixing at compartmental boundaries in *Drosophila* embryos. Nat Cell Biol *12*, 60–65, sup pp 61-69.

Mummery-Widmer, J.L., Yamazaki, M., Stoeger, T., Novatchkova, M., Bhalerao, S., Chen, D., Dietzl, G., Dickson, B.J., and Knoblich, J.A. (2009). Genome-wide analysis of Notch signalling in *Drosophila* by transgenic RNAi. Nature *458*, 987–992.

Nakajima, Y., Meyer, E.J., Kroesen, A., McKinney, S.A., and Gibson, M.C. (2013). Epithelial junctions maintain tissue architecture by directing planar spindle orientation. Nature *500*, 359–362.

Narumiya, S., and Yasuda, S. (2006). Rho GTPases in animal cell mitosis. Curr. Opin. Cell Biol. *18*, 199–205.

Niiya, F., Tatsumoto, T., Lee, K.S., and Miki, T. (2006). Phosphorylation of the cytokinesis regulator ECT2 at G2/M phase stimulates association of the mitotic kinase Plk1 and accumulation of GTP-bound RhoA. Oncogene *25*, 827–837.

O'Keefe, L., Somers, W.G., Harley, A., and Saint, R. (2001). The pebble GTP exchange factor and the control of cytokinesis. Cell Struct. Funct. *26*, 619–626.

Oceguera-Yanez, F., Kimura, K., Yasuda, S., Higashida, C., Kitamura, T., Hiraoka, Y., Haraguchi, T., and Narumiya, S. (2005). Ect2 and MgcRacGAP regulate the activation and function of Cdc42 in mitosis. J. Cell Biol. *168*, 221–232.

Petronczki, M., Glotzer, M., Kraut, N., and Peters, J.M. (2007). Polo-like kinase 1 triggers the initiation of cytokinesis in human cells by promoting recruitment of the RhoGEF Ect2 to the central spindle. Dev. Cell *12*, 713–725.

Prokopenko, S.N., Brumby, A., O'Keefe, L., Prior, L., He, Y., Saint, R., and Bellen, H.J. (1999). A putative exchange factor for Rho1 GTPase is required for initiation of cytokinesis in *Drosophila*. Genes Dev. *13*, 2301–2314.

Prokopenko, S.N., Saint, R., and Bellen, H.J. (2000). Tissue distribution of PEBBLE RNA and pebble protein during *Drosophila* embryonic development. Mech. Dev. *90*, 269–273.

Reinsch, S., and Karsenti, E. (1994). Orientation of spindle axis and distribution of plasma membrane proteins during cell division in polarized MDCKII cells. J. Cell Biol. *126*, 1509–1526.

Ren, G., Vajjhala, P., Lee, J.S., Winsor, B., and Munn, A.L. (2006). The BAR domain proteins: molding membranes in fission, fusion, and phagy. Microbiol. Mol. Biol. Rev. *70*, 37–120.

Riedl, J., Crevenna, A.H., Kessenbrock, K., Yu, J.H., Neukirchen, D., Bista, M., Bradke, F., Jenne, D., Holak, T.A., Werb, Z., et al. (2008). Lifeact: a versatile marker to visualize F-actin. Nat. Methods *5*, 605–607.

Rosenblatt, J., Cramer, L.P., Baum, B., and McGee, K.M. (2004). Myosin II-dependent cortical movement is required for centrosome separation and positioning during mitotic spindle assembly. Cell *117*, 361–372.

Schindelin, J., Arganda-Carreras, I., Frise, E., Kaynig, V., Longair, M., Pietzsch, T., Preibisch, S., Rueden, C., Saalfeld, S., Schmid, B., et al. (2012). Fiji: an open-source platform for biological-image analysis. Nat. Methods *9*, 676–682.

Sedzinski, J., Biro, M., Oswald, A., Tinevez, J.Y., Salbreux, G., and Paluch, E. (2011). Polar actomyosin contractility destabilizes the position of the cytokinetic furrow. Nature *476*, 462–466.

Somers, W.G., and Saint, R. (2003). A RhoGEF and Rho family GTPase-activating protein complex links the contractile ring to cortical microtubules at the onset of cytokinesis. Dev. Cell *4*, 29–39.

St Johnston, D., and Ahringer, J. (2010). Cell polarity in eggs and epithelia: parallels and diversity. Cell *141*, 757–774.

Stewart, M.P., Helenius, J., Toyoda, Y., Ramanathan, S.P., Muller, D.J., and Hyman, A.A. (2011). Hydrostatic pressure and the actomyosin cortex drive mitotic cell rounding. Nature *469*, 226–230.

Su, K.C., Takaki, T., and Petronczki, M. (2011). Targeting of the RhoGEF Ect2 to the equatorial membrane controls cleavage furrow formation during cytokinesis. Dev. Cell *21*, 1104–1115.

Tatsumoto, T., Xie, X., Blumenthal, R., Okamoto, I., and Miki, T. (1999). Human ECT2 is an exchange factor for Rho GTPases, phosphorylated in G2/M phases, and involved in cytokinesis. J. Cell Biol. *147*, 921–928.

Théry, M., Racine, V., Pépin, A., Piel, M., Chen, Y., Sibarita, J.B., and Bornens, M. (2005). The extracellular matrix guides the orientation of the cell division axis. Nat. Cell Biol. *7*, 947–953.

Touré, A., Dorseuil, O., Morin, L., Timmons, P., Jégou, B., Reibel, L., and Gacon, G. (1998). MgcRacGAP, a new human GTPase-activating protein for Rac and Cdc42 similar to *Drosophila* rotundRacGAP gene product, is expressed in male germ cells. J. Biol. Chem. *273*, 6019–6023.

van Impel, A., Schumacher, S., Draga, M., Herz, H.M., Grosshans, J., and Müller, H.A. (2009). Regulation of the Rac GTPase pathway by the multifunctional Rho GEF Pebble is essential for mesoderm migration in the *Drosophila* gastrula. Development *136*, 813–822.

Warner, S.J., and Longmore, G.D. (2009). Cdc42 antagonizes Rho1 activity at adherens junctions to limit epithelial cell apical tension. J. Cell Biol. *187*, 119–133.

Wirtz-Peitz, F., Nishimura, T., and Knoblich, J.A. (2008). Linking cell cycle to asymmetric division: Aurora-A phosphorylates the Par complex to regulate Numb localization. Cell *135*, 161–173.

Zang, J.H., Cavet, G., Sabry, J.H., Wagner, P., Moores, S.L., and Spudich, J.A. (1997). On the role of myosin-II in cytokinesis: division of *Dictyostelium* cells under adhesive and nonadhesive conditions. Mol. Biol. Cell *8*, 2617–2629.

Zavortink, M., Contreras, N., Addy, T., Bejsovec, A., and Saint, R. (2005). Tum/RacGAP50C provides a critical link between anaphase microtubules and the assembly of the contractile ring in *Drosophila melanogaster*. J. Cell Sci. *118*, 5381–5392.

Zhu, X., Wang, J., Moriguchi, K., Liow, L.T., Ahmed, S., Kaverina, I., and Murata-Hori, M. (2011). Proper regulation of Cdc42 activity is required for tight actin concentration at the equator during cytokinesis in adherent mammalian cells. Exp. Cell Res. *317*, 2384–2389.

Developmental Cell

Supplemental Information

Ect2/Pbl Acts via Rho and Polarity Proteins to Direct the Assembly of an Isotropic Actomyosin Cortex upon Mitotic Entry

André Rosa, Evi Vlassaks, Franck Pichaud, and Buzz Baum

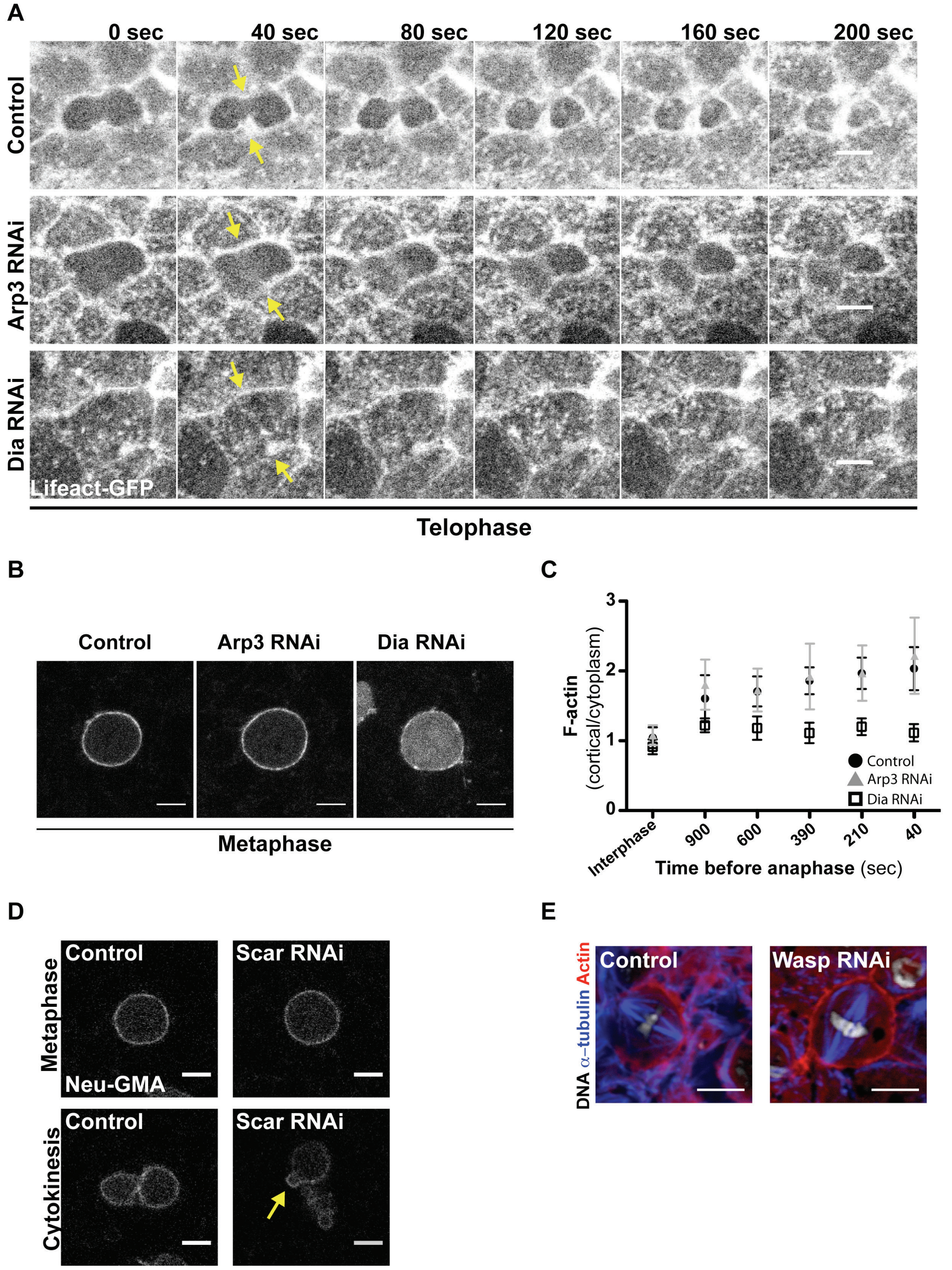

Figure S1. The Arp2/3 complex is not required for the assembly of a mitotic actin cortex; Related to Fig.2. (A) Time-lapse shows the apical portion of a cell expressing UAS-Lifeact::GFP together with *arp3* or *dia* dsRNA imaged in the plane of the epithelium. Time T=0 sec indicates the

onset of cytokinesis. Yellow arrow points to furrowing in control and Arp3 RNAi cells and to the equivalent region of Dia RNAi cells. **(B)** Metaphase cells viewed in the plane of the epithelium expressing UAS-GMA (control) to label F-actin together with Arp3 or Dia RNAi. **(C)** Graph indicates the cortical/cytoplasm ratio of F-actin intensity in the spindle plane as control, Arp3 and Dia RNAi cells enter mitosis. (Mean±SD, N≥25 cells from at least 3 different pupae). **(D)** The cortex of control (Neu:GMA) and SCAR RNAi cells in mitosis and following mitotic exit imaged at the level of the spindle in xy. **(E)** Control and Wasp RNAi cells imaged in the plane of the epithelium stained for filamentous actin (red), DNA (white) and α-tubulin (blue). Scale bar: 5 μm.

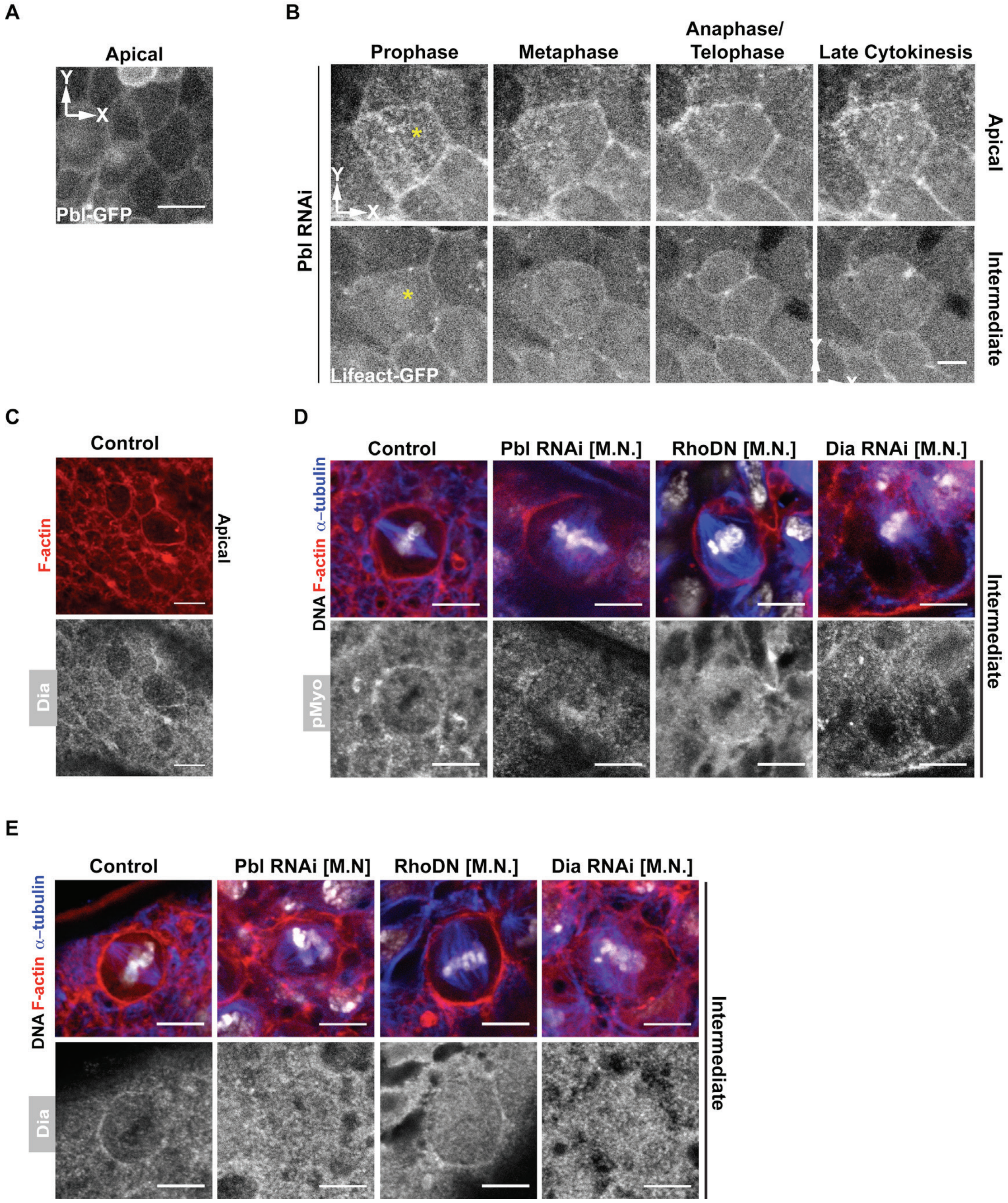

Figure S2. Pbl/Ect2 is required for the correct cortical localization of Dia and Myosin-II; Related to Fig.3. (A) An apical section showing interphase epithelial cells expressing low levels of UAS-Pbl::GFP imaged in the plane of the epithelium. **(B)** Characteristic hypomorphic Pbl RNAi phenotype in a cell labelled with UAS-Lifeact::GFP (as seen in Figure 3) imaged at apical and intermediate sections in the plane of the epithelium. Yellow asterisk indicates a dividing cell. **(C)** Apical section of tissue to show the localization of F-actin (red) and Diaphanous (white) during interphase. **(D)** F-actin (red), DNA

(white), α-tubulin (blue) and phospho-Myosin-II (white) in control, Pbl RNAi, Dia RNAi and Rho1.N19 expressing cells at metaphase. **(E)** As for D, but with Diaphanous shown in white. M.N. = Multinucleated. Scale bar: 5 μm.

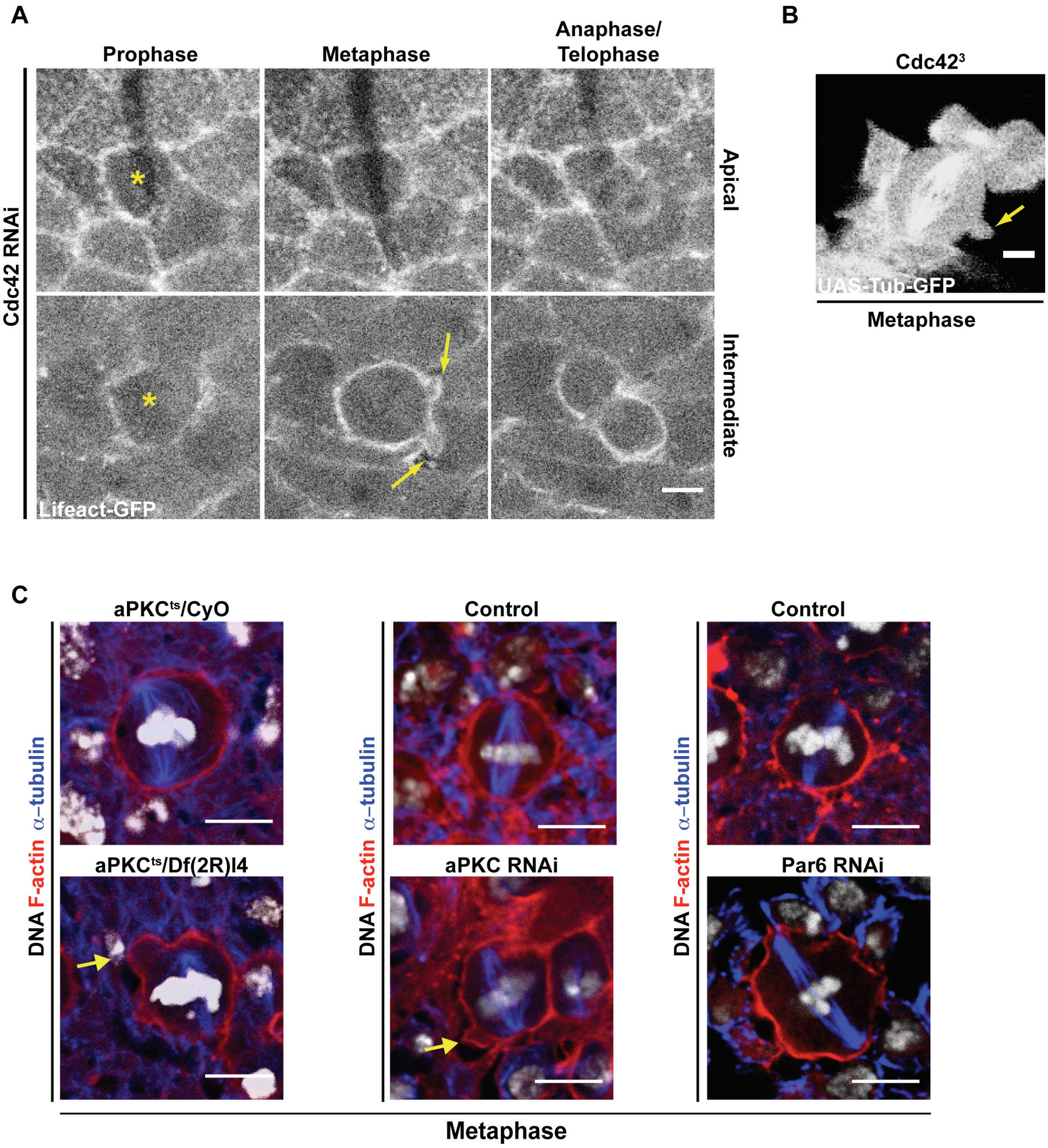

Figure S3. Cdc42, aPKC and Par6 control actin cortex stability; Related to Fig.4. (A) Apical and spindle-level (intermediate) views in the plane of the epithelium for Cdc42 RNAi cells expressing UAS-Lifeact::GFP at different stages of mitosis (as seen in Figure 4). Yellow asterisk indicates dividing cell. Yellow arrows indicate blebbing. **(B)** Mosaic clone of marked *cdc42*^{3} cells expressing UAS-Tub::GFP viewed in xy. Yellow arrow indicates metaphase bleb. **(C)** Control, aPKC RNAi, Par6 RNAi and homozygous *aPKC*^{ts} mutant cells in metaphase labelled to show F-actin (red), DNA (white) and α-tubulin (blue). Yellow arrows indicate cell shape deformations. Scale bar: 5 μm.

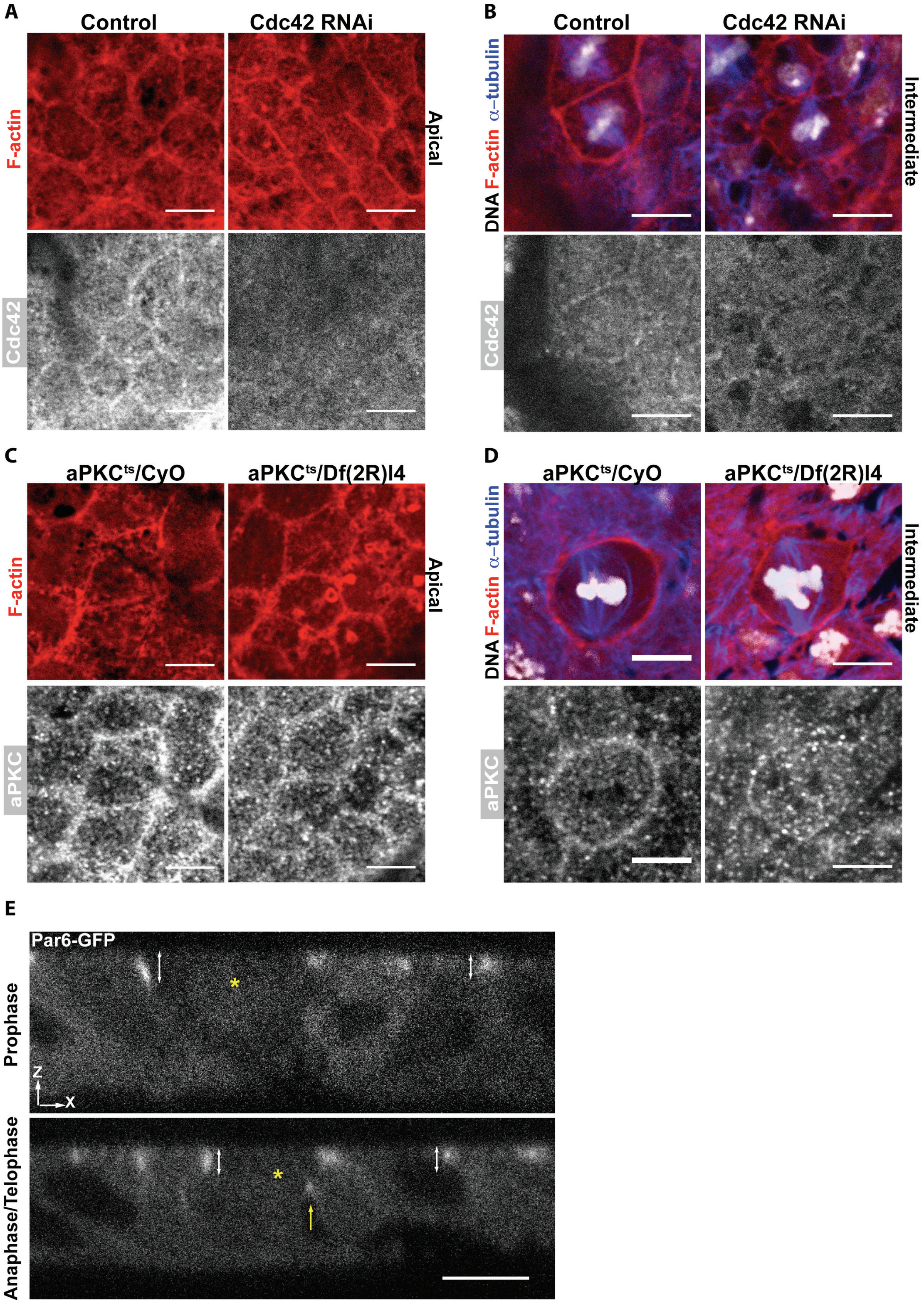

Figure S4. Lateral spreading of Cdc42, aPKC and Par6 during mitosis; Related to Fig.5. (A) Cdc42 RNAi cells labelled for F-actin (red) and using anti-Cdc42 antibody (white) imaged in xy at an apical plane of the epithelium. **(B)** A mitotic Cdc42 RNAi cell stained for F-actin (red), DNA (white), α-tubulin (blue) and Cdc42 (white). **(C)** Control *(aPKC^{ts}/CyO)* and homozygous *aPKC^{ts}*

mutant tissue *(aPKC^{ts}/Df(2R)l4)* labelled for F-actin (red) and aPKC (white) and imaged in xy at the apical plane of the tissue. **(D)** As in C but stained for F-actin (red), DNA (white), α-tubulin (blue) and aPKC (white) to show mitotic cells in xy at the spindle plane. **(E)** Live imaging of a cell expressing Par6-GFP at prophase and anaphase/telophase in cross-section (xz). White double arrow marks polarized interphase Par6::GFP. Yellow asterisk marks dividing cell. Yellow arrow marks cleavage furrow. Scale bar: 5μm.

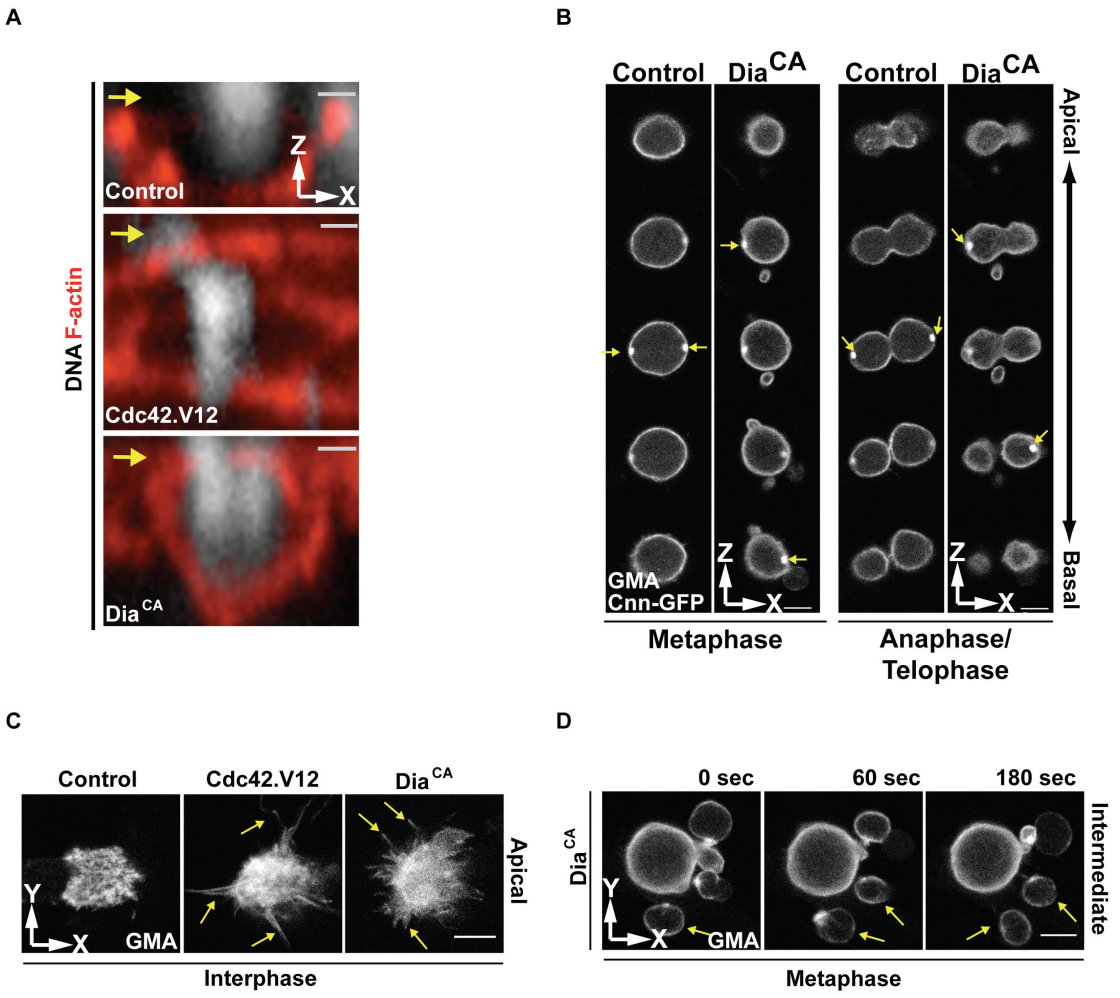

Figure S5. Constitutively active forms of Cdc42 and Dia are sufficient to drive apical filament formation in mitosis; Related to Fig.6. (A) Cross section of control, Cdc42.V12 and Dia^{CA} expressing cells in metaphase labelled for F-actin (red) and DNA (white). Note apical actin-rich cortex in Cdc42.V12 and Dia^{CA} cells (yellow arrow). Scale bar: 1.3 µm. **(B)** Confocal sections of plane of the tissue (xy) taken from apical to basal for control and Dia^{CA} expressing SOP cells marked with UAS-GMA::GFP and UAS-CNN::GFP in metaphase and telophase. Yellow arrows mark centrosomes. Scale bar: 5 µm. **(C)** Apical section in plane of epithelium (xy) of actin in control, Cdc42.V12 and Dia^{CA} expressing SOP cells in interphase marked with UAS-GMA::GFP. Yellow arrows mark actin-rich protrusions. Scale bar: 5 µm. **(D)** Time-lapse movie of a mitotic Dia^{CA} expressing SOP cell imaged in xy at the level of the spindle using UAS-GMA::GFP. Yellow arrows mark blebs that have been pinched off. Scale bar: 5 µm.

Supplemental Experimental Procedures

Fly Stocks

RNAi lines used to silence the expression of the following genes: *cdc42* (NIG-Fly library, ID 12530R-3), *aPKC* (VDRC-Fly library, ID 105624), *arp3* (VDRC-Fly library, ID 35260), *diaphanous* (VDRC-Fly library, ID 103914), *pebble* (VDRC-Fly library, ID 109305), *par-6* (VDRC-Fly library, ID 19731).

Dissections and live imaging

The expression of Neu-GMA was used to label actin filaments in P1 cells (Cohen et al., 2010); UAS-Tub::RFP was expressed from the *pnr* promoter to label microtubules in epithelial cells; UAS-Lifeact::GFP was expressed from the *pnr* promoter to label actin filaments in epithelial cells. The Neu-Gal4 driver was used to express UAS-CNN::GFP, UAS-GMA and UAS-Dia^{CA} in P1 cells. RNAi-induced gene silencing was accomplished by using the *pnr*-Gal4 driver to express Gal4-responsive hairpin dsRNAs in transgenic flies (Mummery-Widmer et al., 2009). In case of RhoDN, Cdc42.V12 and Dia^{CA} transgenes and where the RNAi transgenes were toxic, the Gal80ts system was used to limit expression, after which flies were shifted to 29°C at 6-9 hours AP (Zeidler et al., 2004).

$apkc^{ts}$ zygotic mutant pupae were obtained by crossing the $apkc^{ts}$/CyO, pAct5C-GFP stock with the Df(2R)l4/CyO, pAct5C-GFP and pupae were selected by the absence of GFP, whereas GFP-positive larvae were used as controls.

The following antibodies and dyes were used at the indicated dilutions for our study: Guinea Pig anti-Par6 1:500 (gift from Frank Pichaud); Rabbit anti-

aPKCζ (c-20) 1:100 (Santa Cruz Biotechonology); Rabbit pMyosin Light Chain II (S19) 1:30 (Cell Signaling Technology); Mouse anti-α-tubulin (clone DM1A) 1:250 (Sigma); Rabbit anti-Cdc42 1:20 (Eurogentec); Chicken anti-GFP 1:500 (Abcam); rabbit anti-V5 1:100 (Abcam); Rabbit anti-Dia 1:400 (Gift from Steven A. Wasserman); TRITC-conjugated Phalloidin 1:500; DAPI 1:1000. Secondary antibodies from Molecular Probes were labeled with Alexa 488, 546 and 647 dyes.

Expression vectors, Cell culture and Immunoprecipitation

The Par6 and Pbl cDNA constructs were generated through the Gateway system (Invitrogen) using full-length Drosophila Par-6 and Pbl cDNA as template. Fragments were inserted in frame into the pAct5C vector (DGRC, Indiana University) to create C-terminal Flag-tagged and N-terminal HA-tagged constructs respectively. For transfection, cells were seeded at $2x10^6$ cells / 600 μl in a 12 well plate and allowed to adhere overnight.

48h after transfection (Effectene, Qiagen), whole cell lysates were washed once with PBS and homogenized in lysis buffer (50mM Tris pH7.5, 150mM NaCl, 1% Triton X-100, 1mM EDTA) containing protease (Roche) and phosphatase (Sigma) inhibitor cocktail. Following centrifugation, 500 μg of supernatant was immunoprecipitated with 5 μg of antibody (rabbit anti-HA [abcam], rat anti-HA [Roche] or mouse anti-flag [Sigma]) in 500 μl lysis buffer overnight at 4°C on a rotator. Protein A/G magnetic beads (Pierce, Thermo Scientific) were added, and the mixture was incubated for an additional 1h at 4°C. Beads/extracts were washed three times with BSA/lysis buffer solution followed by three washes in lysis buffer. Bound proteins were eluted from the

beads in 2x Laemmli buffer by boiling for 10 min before separation by SDS-PAGE. For Western Blot analysis, gels were blotted to a polyvinylidene difluoride membrane. Blots were then incubated in 5% milk for 30 min in PBS-0.1%Tween-20, in primary antibody (rabbit anti-HA [abcam], mouse anti-flag [Sigma] or rabbit anti-aPKC [Sigma]) overnight at 4°C, and finally in secondary antibody for 1h.

In vitro binding assay

Recombinant GST::Cdc42 was produced in BL21-A1 *E. coli* by using the pDEST-15 vector (Invitrogen). 4 h after induction with 0.2% L-arabinose, cells were pelleted and resuspended in GST lysis buffer [50 mM Tris–HCl pH 7.6, 50 mM NaCl, 5mM $MgCl_2$, 10 mM dithiothreitol (DTT), protease inhibitor cocktail Complete (Roche)]. Cells were sonicated and debris was removed by centrifugation at 9000 RPM for 15 min. Glutathione–Sepharose beads were incubated with the supernatant on a rotator for 30 min at 4°C. Beads were washed 5 times with GST storage buffer [50 mM Tris–HCl pH 7.6, 50 mM NaCl, 5mM $MgCl_2$, 1 mM dithiothreitol (DTT), protease inhibitor cocktail Complete (Roche)], and stored in the same buffer. Production and purification of GST::Cdc42 proteins were verified by Coomassie staining of SDS/PAGE gel. GST-pull-down assays were performed by using 300 µl of Myc::Pbl transfected (or salmon sperm transfected as negative control) S2 cell lysates along with 5-30 µl of glutathione-Sepharose beads with bound GST-Cdc42. After 1h incubation with rotation at 4°C, the supernatant was removed and the beads were washed five times with lysis buffer (see immunoprecipitation for recipe). After SDS/PAGE, Pbl and recombinant GST proteins were detected

by Western blotting using anti-Myc (Santa Cruz) and anti-GST (Sigma) antibody.

References

Cohen, M., Georgiou, M., Stevenson, N.L., Miodownik, M., and Baum, B. (2010). Dynamic filopodia transmit intermittent Delta-Notch signaling to drive pattern refinement during lateral inhibition. Developmental cell *19*, 78-89.

Mummery-Widmer, J.L., Yamazaki, M., Stoeger, T., Novatchkova, M., Bhalerao, S., Chen, D., Dietzl, G., Dickson, B.J., and Knoblich, J.A. (2009). Genome-wide analysis of Notch signalling in Drosophila by transgenic RNAi. Nature *458*, 987-992.

Zeidler, M.P., Tan, C., Bellaiche, Y., Cherry, S., Hader, S., Gayko, U., and Perrimon, N. (2004). Temperature-sensitive control of protein activity by conditionally splicing inteins. Nature biotechnology *22*, 871-876.
